# The mathematical influence on global patterns of biodiversity

**DOI:** 10.1002/ece3.6385

**Published:** 2020-06-11

**Authors:** Gregory Beaugrand, Richard Kirby, Eric Goberville

**Affiliations:** ^1^ LOG Laboratoire d'Océanologie et de Géosciences CNRS UMR 8187 Wimereux France; ^2^ The Secchi Disk Foundation Plymouth UK; ^3^ Unité Biologie des Organismes et Ecosystèmes Aquatiques (BOREA) Muséum National d’Histoire Naturelle Sorbonne Université Université de Caen Normandie Université des Antilles CNRS IRD Paris France

**Keywords:** biodiversity, ecological niche, large‐scale patterns in species richness, models, theory

## Abstract

Although we understand how species evolve, we do not appreciate how this process has filled an empty world to create current patterns of biodiversity. Here, we conduct a numerical experiment to determine why biodiversity varies spatially on our planet. We show that spatial patterns of biodiversity are mathematically constrained and arise from the interaction between the species’ ecological niches and environmental variability that propagates to the community level. Our results allow us to explain key biological observations such as (a) latitudinal biodiversity gradients (LBGs) and especially why oceanic LBGs primarily peak at midlatitudes while terrestrial LBGs generally exhibit a maximum at the equator, (b) the greater biodiversity on land even though life first evolved in the sea, (c) the greater species richness at the seabed than at the sea surface, and (d) the higher neritic (i.e., species occurring in areas with a bathymetry lower than 200 m) than oceanic (i.e., species occurring in areas with a bathymetry higher than 200 m) biodiversity. Our results suggest that a mathematical constraint originating from a fundamental ecological interaction, that is, the niche–environment interaction, fixes the number of species that can establish regionally by speciation or migration.

## Graphical abstract

We show that spatial patterns of biodiversity are mathematically constrained and arise from the interaction between the species' ecological niches and environmental variability that propagates to the community level. Our results allow us to explain key biological observations such as (a) latitudinal biodiversity gradients in the terrestrial and the marine (surface and bottom) domains, (b) the greater biodiversity on land even though life first evolved in the sea, (c) the greater species richness at the seabed than at the sea surface, and (d) higher neritic than oceanic biodiversity.

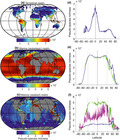

## 1 INTRODUCTION

One of the most fundamental curiosities in biology is to understand what influences biodiversity and its spatial and temporal distribution (Gaston, 2000; Lomolino, Riddle, & Brown, 2006). Currently, biologists have described 1,233,500 species on land and 193,756 in the sea with recent estimates of the total number of species in these realms suggested to be 8,740,000 (terrestrial) and 2,210,000 (marine) (Mora, Tittensor, Adl, Simpson, & Worm, 2011). Clearly, biodiversity is not uniformly distributed between land and sea. Moreover, on land and in the sea, many taxonomic groups exhibit a latitudinal increase in species richness from the poles to the midlatitudes or the equator (Gaston, 2000; Lomolino et al., 2006; Tittensor et al., 2010). What causes these latitudinal gradients in species richness has been a topic of study and debate for decades (Rosenzweig & Sandlin, 1997), and more than 25 hypotheses have now been proposed (Gaston, 2000).

Neo‐Darwinism predicts that natural selection favors the fittest genetic composition, and we know that genetic isolation can lead to speciation to progressively fill vacant niches (Gould, 1977). However, neither natural selection nor speciation alone can explain (a) why there are more species on land than in the sea, (b) why there are different latitudinal biodiversity gradients (LBGs) exhibited on land (narrow maximum at the equator) and in the ocean (maximum observed over midlatitudes with sometimes a small diminution at the equator), (c) why the sea exhibits greater biodiversity on the seabed than in the pelagic zone, and (d) why there are more (pelagic and benthic) neritic (i.e., continental‐shelf species, species occurring in areas lower than 200 m) than oceanic (i.e., species occurring in areas higher than 200 m) species.

Here, we conduct numerical experiments to show that these biological observations can be explained by a mathematical constraint on the arrangement of life that originates from a fundamental interaction, that is, the niche–environment interaction. Our results suggest that this mathematical constraint fixes the maximum number of species that can establish regionally.

## 2 DATA

### 2.1 Land surface climatic data

Mean monthly temperature (°C) and precipitation (mm) climatologies (period 1970–2000) were retrieved from the 1‐km spatial resolution WorldClim version 2 dataset (http://worldclim.org/version2; Fick & Hijmans, 2017). Climatologies were obtained by performing the thin‐plate smoothing spline algorithm implemented in the ANUSPLIN package; more information on the numerical procedures is available in Hijmans, Cameron, Parra, Jones, and Jarvis (2005) and Fick and Hijmans (2017). Temperature and precipitation data were linearly interpolated monthly on a grid of 0.25° × 0.25° (for simulations without considering the potential influence of allopatric speciation) and 2° × 2° (for simulations considering the potential influence of allopatric speciation).

Mean monthly sea‐level pressures (SLP) and downward solar radiation at surface originated from ERA‐Interim from the European Centre for Medium‐Range Weather Forecasts (ECMWF; Berrisford et al., 2011). A climatology (period 1979–2012) was calculated on a spatial grid 0.5° × 0.5° and was used to estimate the relationships between biodiversity patterns and atmospheric processes.

### 2.2 Marine hydroclimatic data and bathymetry

Monthly sea surface temperature (SST) originated from weekly optimum interpolation (OI SST v2; 1982–2017). Monthly SST is commonly used as a proxy of the temperature experienced in the epipelagic zone (Beaugrand, Edwards, & Legendre, 2010; Tittensor et al., 2010).

We used temperature (°C) and light (E m^−2^ year^−1^) at the seabed from the Bio‐ORACLE v2.0 initiative (http://www.bio‐oracle.ugent.be; Assis et al., 2017; Tyberghein et al., 2012), a comprehensive set of 23 geophysical, biotic, and climate data layers for present (2000–2014) conditions, statistically downscaled (i.e., from coarse‐ to fine‐scale resolution) to a common spatial resolution of 5 arcmin (9.2 km at the equator). Further descriptions of the layers, data sources, and quality control maps can be found on the Bio‐ORACLE Web site and the literature (Assis et al., 2017; Tyberghein et al., 2012). Monthly temperature and light were linearly interpolated on a grid of 0.25° × 0.25° (for simulations without consideration of the potential influence of allopatric speciation) and 2° × 2° (for simulations with consideration of the potential influence of allopatric speciation). Light was used as a filter for benthic species that need light at the seabed (e.g., coral reef, mangrove, and seagrass).

Bathymetry data were extracted from the General Bathymetric Chart of the Ocean (GEBCO; www.gebco.net/data_and_products/gridded_bathymetry_data).

### 2.3 Biological data

The dataset of observed biodiversity for the marine realm was provided by Dr Derek Tittensor, Dalhousie University (Tittensor et al., 2010). The data were compiled from empirical sampling data (foraminifers and bony fish) or from expert‐verified range maps encompassing many decades of records. The data were originally gridded on a 880‐km equal‐area resolution grid (Tittensor et al., 2010). We used all data but pinnipeds, which showed an inverse LBG that we explained in our previous studies (Beaugrand, Luczak, Goberville, & Kirby, 2018; Beaugrand, Rombouts, & Kirby, 2013) by the place of origination of the taxon. The seven neritic groups were seagrasses, mangroves, corals, non‐oceanic sharks, coastal fishes, non‐squid, and squid cephalopods, and the five oceanic groups were foraminifera, euphausiids, oceanic sharks, tunas and billfishes, and cetaceans. Note that non‐squid and squid cephalopods were classified as primarily neritic on the basis of the examination of figure 1 in Tittensor et al. (2010).

We obtained terrestrial realm biodiversity datasets from published and freely available sources including the web‐based platform Data Basin (http://www.databasin.org) managed by the Conservation Biology Institute (CBI) and the BiodiversityMapping.org Web site developed by Clinton Jenkins (Jenkins, Pimm, & Joppa, 2013; Pimm et al., 2014). Terrestrial variables were plants, amphibians, lizards and snakes, turtles and crocodilians, reptiles, birds (including breeding and non‐breeding species), and mammals. These datasets (available as GIS layers) were originally gridded (a) at the eco‐regional scale when provided by the CBI and (b) on a 10 × 10 km grid using the Eckert IV equal‐area projection for data originated from Biodiversity Mapping. Detailed descriptions of each dataset and information about the methods applied to generate the layers are available at http://maps.tnc.org/globalmaps.html (Hoekstra et al., 2010) and at https://biodiversitymapping.org/wordpress/index.php/download/, respectively.

## 3 METHODS

### 3.1 The macroecological theory on the arrangement of life

The MacroEcological Theory on the Arrangement of Life (METAL) is a theory that explains how life is arranged and how changing environmental conditions alter biological arrangements in space and time at different organizational levels (e.g., species, community, ecosystem), allowing precise predictions to be tested. The METAL theory, described in more details in Text S1, postulates that many ecological (e.g., phenology, annual plankton succession), biogeographic (e.g., LBGs), and climate‐change biology patterns (e.g., phenological and biogeographic shifts) originate from the fundamental niche–environment interaction (Beaugrand, 2015a, 2015b; Beaugrand et al., 2013, 2018, 2019; Beaugrand, Edwards, Raybaud, Goberville, & Kirby, 2015; Beaugrand, Goberville, Luczak, & Kirby, 2014; Beaugrand & Kirby, 2018b). The METAL theory unifies a large number of patterns observed in biogeography and ecology at different organizational levels (e.g., spatial range, Rapoport's rule, phenology, annual plankton succession, latitudinal biodiversity gradients, formation and alteration of species assemblages) and in climate‐change biology (e.g., phenological shifts, year‐to‐year to decadal changes in species abundance, range shift, biodiversity shifts, community alteration, abrupt community shifts; Beaugrand, 2015a, 2015b, 2019; Beaugrand et al., 2013, 2014, 2015, 2018; Beaugrand & Kirby, 2016, 2018b).

The theory uses the concept of the ecological niche sensu Hutchinson (Hutchinson, 1957) as a macroscopic elementary ‘brick’ to understand how species fluctuate in time and space and how communities form and are altered by environmental fluctuations, including climate change. All species have an ecological niche, which means that they operate within a range of ecological conditions that are suitable for growth and reproduction. The environment acts by selecting species that have the appropriate niche. It follows that this mechanism determines the place where a species lives (i.e., spatial distribution), time when it is active (i.e., phenology), and how individual density fluctuates from short to long time scales. Locally however, the absence of a species may be explained by species interactions and random processes, such as those discussed in the Unified Neutral Theory of Biodiversity and Biogeography (Hubbell, 2001). The ecological niche, measured by the abundance plotted as a function of some key ecological factors throughout the spatial range of a species, integrates all its genetic variation. More information on the METAL theory can be found in Beaugrand (Beaugrand, 2015a; see also Text S1).

### 3.2 Summary of the approach

Some models have been proposed as part of the METAL theory (Beaugrand et al., 2013, 2014, 2015; Beaugrand & Kirby, 2018a). Here, the model was specifically designed to implement a set of basic ecological/climatic principles to test whether latitudinal gradients in species diversity might arise from the interaction between the ecological niches of species and spatiotemporal (i.e., monthly time scale) fluctuations in temperature and/or precipitation related to climate variability. The model has been fully described and tested in Beaugrand and colleagues (Beaugrand et al., 2013, 2018).

The principle of the model is simple. It starts to create a large number of niches on the basis of temperature only (marine realm) or using both temperature and precipitation (terrestrial realm). In a given area, each pseudo‐species has a unique niche after the principle of competitive exclusion of Gause (1934) while considering niche overlapping (Beaugrand et al., 2013, 2015). METAL models have been tested for marine taxonomic groups for which species's realized ecological niches were assessed. Correlations between biodiversity estimated from modeled species distribution and biodiversity assessed from METAL were highly significant (G. Reygondeau, personal communication).

Two main numerical experiments were conducted. In the first set of experiments conducted at a spatial resolution of 0.25° latitude × 0.25° longitude, species were allowed to colonize a given oceanic region so long as they could tolerate changes in the environmental regime at different temporal scales (here at a monthly temporal scale). By reconstructing pseudocommunities, we were able to reproduce the spatial arrangement of biodiversity. In these experiments (a total of twelve, Table 1), the potential for allopatric speciation was not considered and a niche, in a given area, was only occupied by one pseudo‐species. Values of the different parameters (Table 1) were fixed on the basis of 74 in silico experiments carried out in a previous study (Beaugrand et al., 2013).

**TABLE 1:** Values of the different parameters for each simulation

	Simulations	*t*_min_	*t*_max_	*µ^t^*	*s^t^*	*p*_min_	*p*_max_	*µ^p^*	*s^p^*	*R*
Land (no speciation ‐1‐/ speciation ‐2‐)	T & P	−1.8	44	0.1	0.1	0	3,000	100	50	94,299,210
Land (speciation ‐3‐)	T & P	−1.8	44	0.1	0.1	0	3,000	400	200	7,300,584
Land (no speciation ‐4‐/ speciation ‐5‐)	T	−1.8	44	0.1	0.1	—	—	—	—	101,397
Land (no speciation ‐6‐/speciation ‐7‐)	P	—	—	—	—	0	3,000	100	50	930
Land (speciation ‐8‐)	P	—	—	—	—	0	3,000	400	200	72
Surface ocean (all pelagic) (no speciation ‐9‐/speciation ‐10‐)	SST	−1.8	44	0.1	0.1	—	—	—	—	101,397
Surface ocean (nerito‐pelagic) (no speciation ‐11‐/speciation ‐12‐)	SST	−1.8	44	0.1	0.1	—	—	—	—	101,397
Surface ocean (holo‐pelagic) (no speciation ‐13‐/speciation ‐14‐)	SST	−1.8	44	0.1	0.1	—	—	—	—	101,397
Seabed (all benthic) (no speciation ‐15‐/speciation ‐16‐)	T	−1.8	44	0.1	0.1	—	—	—	—	101,397
Seabed (0−200 m) (no speciation with ‐17‐ and without ‐18‐ light at seabed/speciation without light at seabed ‐19‐)	T	−1.8	44	0.1	0.1	—	—	—	—	101,397
Seabed (200−2,000 m) (no speciation ‐20‐/speciation ‐21‐)	T	−1.8	44	0.1	0.1	—	—	—	—	101,397
Seabed (>2,000 m) (no speciation ‐22‐/speciation ‐23‐)	T	−1.8	44	0.1	0.1	—	—	—	—	101,397

Note

A total of 23 simulations were carried out. In the oceanic domain, the values of the parameters were identical when simulations were performed with (2° × 2° spatial resolution) and without (0.25° × 0.25° spatial resolution) consideration for allopatric speciation. This was not the case for land however, where different values were considered because of the high number of calculations involved when considering allopatric speciation. A further simulation was made by considering light at seabed for regions shallower than 200 m at a spatial resolution of 0.25° latitude × 0.25° longitude (see Section 2). Values of *t*_min_ and *t*_max_ were minimal and maximal temperature for niche creation. Similarly, *p*_min_ and *p*_max_ were minimal and maximal precipitation for niche creation.

*µ^t^* and *µ^p^* were values of the step for niche amplitude with respect to temperature and precipitation, respectively. *s^t^* and *s^p^* were the values for niche overlapping with respect to temperature and precipitation, respectively. *R*: total number of niches, *T*: temperature, *P*: precipitation, SST: sea surface temperature, —: not applicable. Units for monthly temperature (*t*_min_, *t*_max_, *u^t^*, and *s^t^*) and precipitation (*p*_min_, *p*_max_, *u^p^*, and *s^p^*) are °C and mm, respectively. Each simulation is numbered.

In the second set of experiments conducted at a spatial resolution of 2° latitude × 2° longitude, we considered the potential for allopatric speciation and eleven simulations were carried out. In these simulations, more than one species could occupy the same niche providing that they were not at the same place, reflecting the first principle of biogeography (Buffon's Law; Lomolino et al., 2006). The potential for allopatric speciation was evaluated when there was a permanent separation between two places at a monthly scale; we did not consider the influence of year‐to‐year to millennium variability. Here, our objective was not to investigate biogeographic cradles, museums and graves but rather to examine the potential influence of allopatric speciation for global patterns of biodiversity and biodiversity difference among realms; the influence of long‐term variability, in addition to evolutive niches, has been recently considered by Rangel and colleagues in a study of the biodiversity in South America (Rangel et al., 2018).

### 3.3 Detailed description of the model and the analyses

**FIGURE 1:**
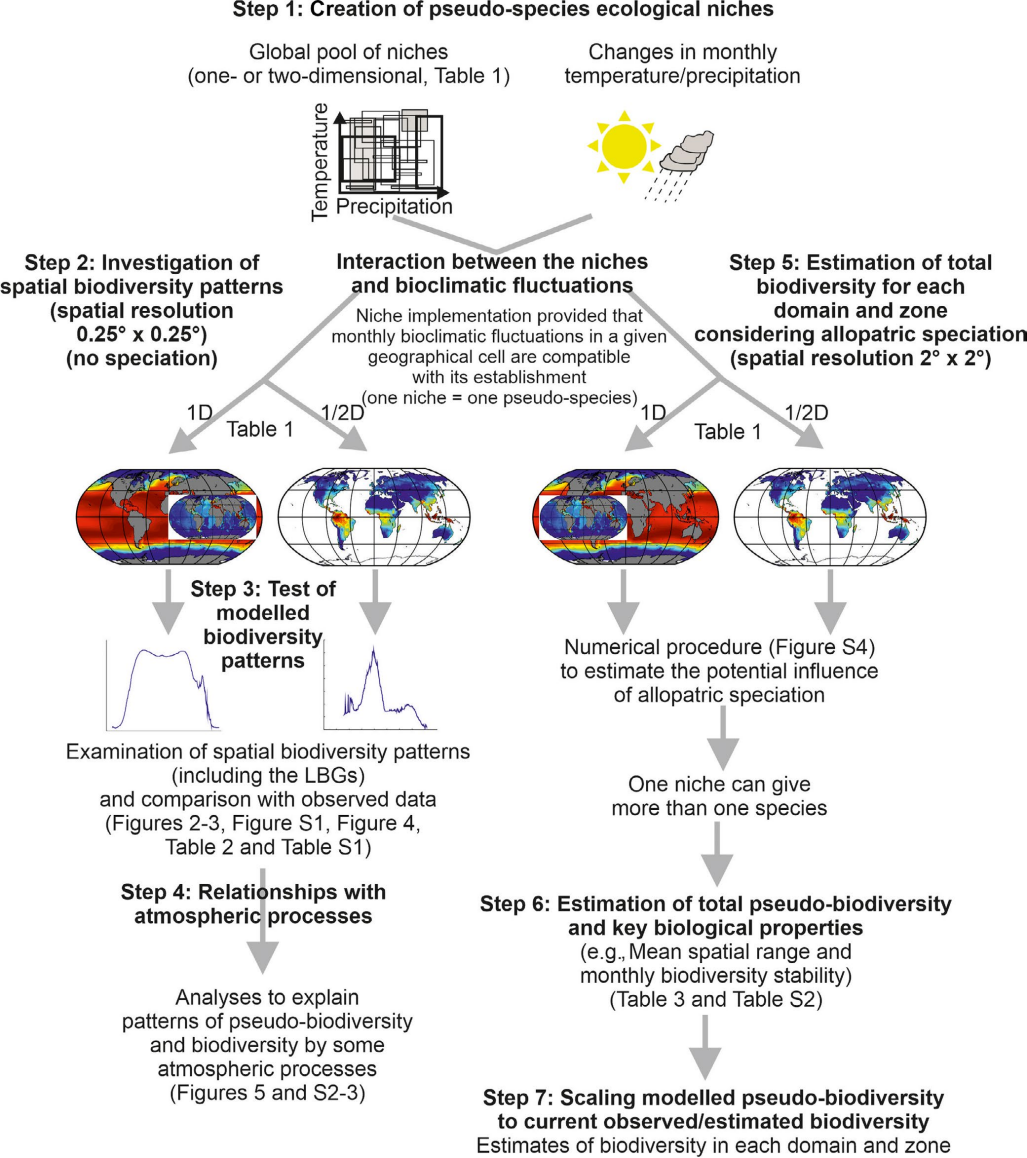
Sketch diagram that summarizes the main numerical analyses performed in this study. D: dimension

An overview of the model and subsequent analyses carried out as part of this study is provided in Figure 1. Simulations and related analyses were performed in seven steps.

#### 3.3.1 Step 1: Creation of species ecological niches

We first created species niches. Following the Hutchinson concept of ecological niche, a niche is defined as the range of tolerance of a species when several environmental parameters are selected simultaneously (Hutchinson, 1957). Here, we reduced the hypervolume of the Hutchinson's niche to a one‐ or two‐dimensional niche by considering only temperature and/or precipitation. We considered this simplification acceptable when we tested the theory in the marine realm as temperature has often been identified as the main controlling factor of pelagic biodiversity patterns (Rombouts et al., 2009; Tittensor et al., 2010) and is known to influence almost all biological processes and systems from individual cells to the whole biosphere (Brown, Gillooly, Allen, Savage, & West, 2004). In the terrestrial domain, considering water availability or precipitation is essential to recreate ecogeographical patterns in diversity and many terrestrial studies have shown there is a synergistic effect of temperature and precipitation on ecosystems (Whittaker, 1975). Because we used species richness as a measure of diversity, the shape of the pseudo‐species’ niche was rectangular (presence/absence), which has the advantage of relaxing the constraint on the shape of the niche (e.g., Gaussian [ter Braak & Prentice, 1988]).

For the marine realm, our model generates a set of pseudo‐species, each being characterized by a specific thermal tolerance. Pseudo‐species, from strict to very large eurytherms, and from psychrophile to more thermophile species were allowed to colonize a given oceanic region so long as they could survive monthly changes in SST (Figure 1). Although we allowed the niche of each pseudo‐species to overlap, we also gave every species a unique niche in a given area after the principle of competitive exclusion (Gause, 1934).

All potential thermal pseudo‐species’ niches ranged from *ρ*_min_ = *t*_min _= −1.8°C to *ρ*_max_ = *t*_max _= 44°C (Table 1). The thermal range was identical on both domains, so no methodological differences between land and ocean occurred. The thermal thresholds were based on Beaugrand et al. (2013): In their paper, several thresholds were used and the consideration of *t*_min _= −1.8°C and *t*_max_ = 44°C gave results strongly correlated with observed biodiversity patterns (see their Table S1). All potential precipitation niches ranged from *ρ*_min_ = *p*_min _= 0 mm to *ρ*_max_ = *p*_max _= 3,000 mm (Table 1). A value for *ρ*_max_, slightly higher than maximum precipitation observed globally for a given month, was chosen. A modification of the maximum precipitation threshold above 3,000 mm did not affect our perception of the LBGs because the maximum of precipitation took place over the equator. An increase in the maximum precipitation threshold only affected the strength of the gradient.

The amplitude *α* of a niche (i.e., the width of a niche) varied between 1°C and 45.8°C for temperature and from 100 mm to 2,900 mm for precipitation by step of *µ* (*µ = µ^t^* for temperature and *µ = µ^p^* for precipitation; Table 1). The amplitude *α* of a niche with respect to temperature or precipitation was calculated as follows:

(1) $$\alpha_{i}=\alpha_{i-1}+\mu \;\text{with}\;2\leq i\leq p$$

With *µ*, the increment between niche amplitudes. *µ^t^* was fixed to 0.1°C for all simulations, and *µ^p^* ranged between 100 mm (simulations with no allopatric speciation) and 400 mm (simulations with allopatric speciation) for precipitation. *α*_1_ = 1°C for temperature and 100 mm for precipitation in all simulations. *p* was the floor value of the quantity:

(2) $$p=\left\lfloor \frac{\alpha_{\max}-\alpha_{1}}{\mu }\right\rfloor +1$$

The maximum amplitude *α*_max_ was calculated as follows:

(3) $$\alpha_{\max}=\rho_{\max}-\rho_{\min}$$

Therefore, *p* varied as a function of both the minimum (*α*_1_) and maximum (*α*_max_) niche amplitude, as well as the increment between niche amplitudes (temperature or precipitation) *µ*; column vector **A** *_p_*_ _= [*α_i_*]. When *α* is large, the niche corresponds to an euryoecious species having the potential to colonize many terrestrial (temperature and/or precipitation) or marine (temperature only) regions. The weight of those euryoecious species in the modeled biodiversity was low, however.

For a given niche amplitude *α_i_* (1 ≤ *i* ≤ *p*), the starting point of a pseudo‐species niche *x* was a function of *ρ*_min_ and *ρ*_max_ and the degree of overlapping between niches *s*, which was fixed to 0.1°C for temperature in all simulations and ranged from 50 mm (simulations with no speciation) to 200 mm (simulations with potential for allopatric speciation) for precipitation. No species had exactly the same niche according to Gause's principle of competitive exclusion (Gause, 1934). For each niche amplitude *α_i_*, the starting point of a pseudo‐species’ niche was calculated as follows:

(4) $$x_{i,j}=x_{i,j-1}+s\;1\leq i\leq p\;2\leq j\leq q_{i}$$

With *x*._1_ = *ρ*_min_. *q_i_* was the floor value of the quantity:

(5) $$q_{i}=\left\lfloor \frac{\alpha_{\max}+s-\alpha_{i}}{s}\right\rfloor +1\;1\leq i\leq p$$

Column vector ***Q*** *_p _*= [*q_i_*]. The ending point of a pseudo‐species’ niche (temperature or precipitation) *y* was determined by adding the niche amplitude to the starting point:

(6) $$y_{i,j}=x_{i,j}+\alpha_{i}\;1\leq i\leq p\;1\leq j\leq q_{i}$$

A total of *r* pseudo‐species was created:

(7) $$r=\sum_{i=1}^{p}q_{i}$$

With *p* being calculated in Equation (2). *r* varied in the different scenarios between 72 (simulation based on precipitation only) and 94 million (simulation based on temperature and precipitation) pseudo‐species (Table 1).

When two ecological dimensions were used (land simulations), the total number of pseudo‐species *R* was the result of the multiplication of *r^t^* by *r^p^*:

(8) $$R=r^{t}\cdot r^{p}$$

With *r^t^* and *r^p^* the number of pseudo‐species based on temperature and precipitation, respectively. *R* = *r^t^* when simulations were exclusively based on temperature or *R* = *r^p^* when they were only based on precipitation.

#### 3.3.2 Step 2: Simulations at a 0.25° × 0.25° spatial resolution to examine spatial patterns in biodiversity

We performed eleven simulations at a 0.25° × 0.25° spatial resolution to assess pseudo‐species richness on land and in the marine realm (Table 1). These simulations were performed by assuming that a niche led to a single pseudo‐species for all continents. The absence of consideration for allopatric speciation had no effect on our estimation of local pseudo‐species richness. Three simulations were carried out on land using (a) both precipitation and temperature, (b) temperature only, and (c) precipitation only (simulations 1, 4, and 6 in Table 1).

In the marine realm, we performed eight simulations, three for the pelagic realm (global surface, nerito‐pelagic, and holo‐pelagic, see glossary in Text S2; simulations 9, 11, and 13 in Table 1) and five for the seabed (global, 0–200 m, 200–2,000 m, and >2,000 m; simulations 15, 17, 18, 20, and 22). These simulations were temperature‐based because precipitation mainly influences littoral biodiversity by acting on continental runoffs at a regional scale (Goberville, Beaugrand, Sautour, & Tréguer, 2010; Table 1). Simulation 18 (Table 1) was made to distinguish an additional area with light at the seabed. This distinction was important to test our model with taxonomic groups that require light at the seabed (e.g., coral reef, mangrove, and seagrass; Table 2). For this zone, we weighted pseudo‐species richness *D* by light at the seabed w:

(9) $$D^{*}=w\cdot D$$

*w* was assessed by applying a *β* distribution, as follows:

(10) $$w=v\left(\frac{e_{\max}-e}{e_{\max}-e_{\text{opt}}}\right){\left(\frac{e-e_{\min}}{e_{\text{opt}}-e_{\min}}\right)}^{\left(\frac{e_{\text{opt}}-e_{\min}}{e_{\max}-e_{\text{opt}}}\right)}$$

where *v* = 1, *e*_max_ = 70, *e*_opt_ = 20, and *e*_min_ = 0. Light at the seabed varied from 0 to 33.43 E m^−2^ year^−1^. The use of different values did not affect significantly our results (not shown).

**TABLE 2:** Correlations between simulated and observed species richness on land and in the ocean

Realm	Group	Geographical cell		Latitudinal biodiversity gradient	
		Correlation	Degree of freedom (*n*) (n*, *p* < .05)	Correlation	Degree of freedom (*n*) (n*, *p* < .05)
Terrestrial	Plant	0.7544	63,105 (6)	0.9396	584 (3)
	Amphibian	0.7024	52,994 (7)	0.8774	501 (4)
	Reptile	0.6938	84,198 (7)	0.6674	584 (7)
	Lizard and snake	0.6580	63,105 (8)	0.6955	584 (7)
	Turtle and crocodilian	0.7521	84,378 (6)	0.8070	584 (5)
	Bird	0.7712	76,528 (5)	0.9295	589 (3)
	Non‐breeding bird	0.7871	75,600 (5)	0.8984	589 (3)
	Breeding bird	0.7534	76,150 (6)	0.9239	589 (3)
	Mammal	0.7688	71,183 (5)	0.9560	589 (2)
Marine epipelagic (oceanic and neritic)	Foraminifera	0.8998	601,804 (3)	0.9095	602 (3)
	Euphausiid	0.8251	601,804 (4)	0.8586	602 (4)
	Oceanic shark	0.7756	601,804 (5)	0.8839	602 (3)
	Tuna and billfish	0.8401	601,804 (4)	0.9118	602 (3)
	Mammal (cetacean)	0.7143	601,804 (6)	0.7987	602 (5)
Neritic (benthic with light at seabed)	Seagrass	0.6948	40,087 (7)	0.8549	557 (4)
	Mangrove	0.7107	40,087 (7)	0.8226	557 (4)
	Coral	0.6406	40,087 (8)	0.7875	557 (5)
Neritic (pelagic and benthic)	Squid	0.6224	49,549 (9)	0.3857	571 (25)
	Non‐squid cephalopod	0.7848	49,549 (5)	0.7037	571 (7)
	Non‐oceanic shark	0.8443	49,549 (4)	0.8577	571 (4)
	Coastal fish	0.6797	49,549 (7)	0.6412	571 (8)

Note

Correlations were calculated on the basis of geographical cells (left) and along latitudes (right).

All correlations were significant at the threshold of 0.05. The degree of freedom (*n*) of each correlation is indicated, and n*, in brackets, denotes the degree of freedom needed to maintain a significant relationship at *p* = .05.

The epipelagic zone is a region between 0 and 200 m (surface ocean). The neritic domain is defined here as the region with a bathymetry between 0 and 200 m. The region below 200 m is the oceanic domain.

For all those simulations performed at a 0.25° latitude × 0.25° longitude spatial resolution, a niche led to the establishment of only one pseudo‐species; the pseudo‐species colonized progressively a given region of the ocean or land so long as they could withstand local monthly changes in temperature, precipitation, or both climatic parameters.

#### 3.3.3 Step 3: Test of the modeled spatial biodiversity patterns

**FIGURE 2:**
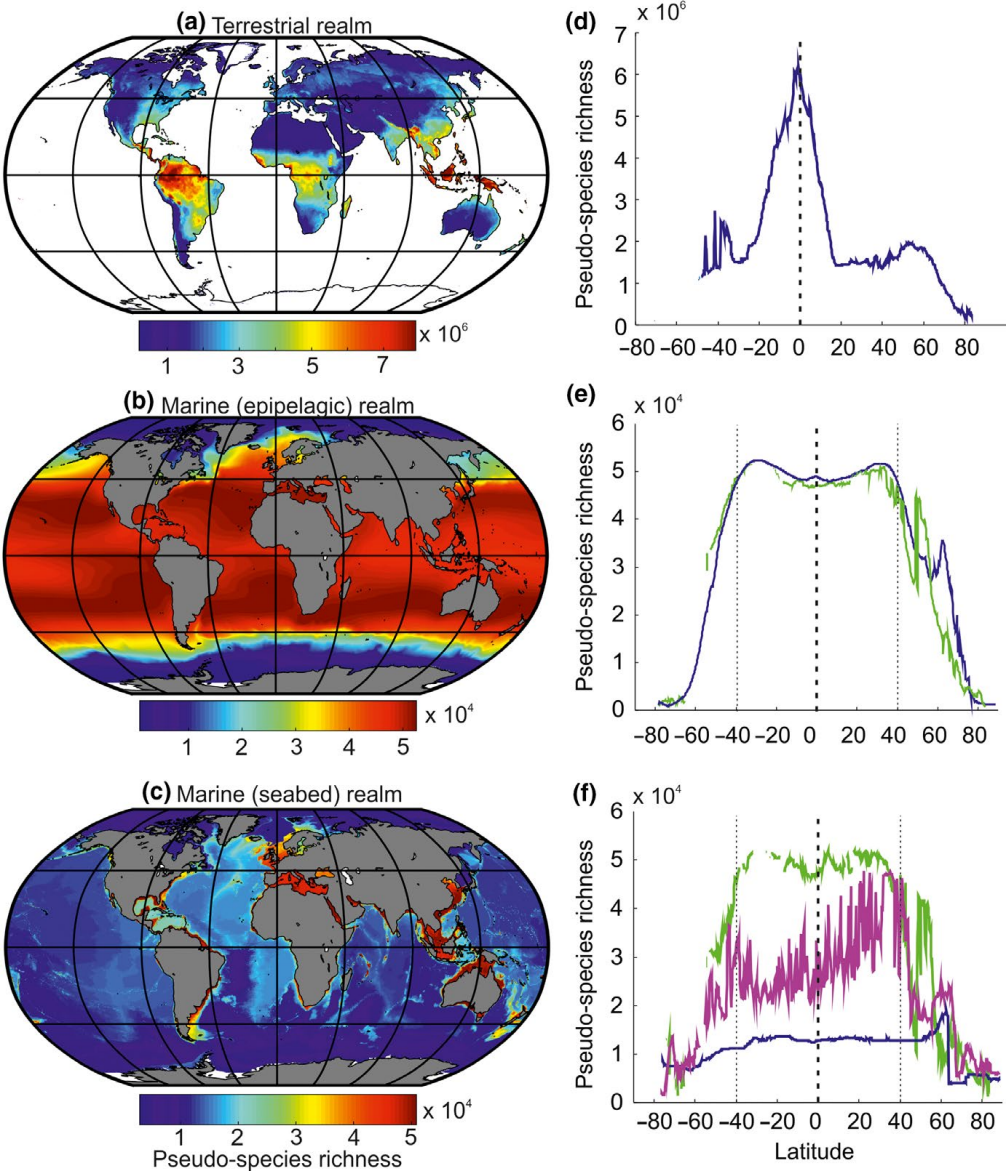
Ecogeographical patterns (a–c) and latitudinal biodiversity gradients (d–f) in pseudo‐species richness. Ecogeographical patterns in pseudo‐species richness in the (a, d) terrestrial, (b, e) the marine epipelagic ocean (bathymetry > 200 m; blue), and the nerito‐pelagic realm (bathymetry < 200 m; green), and the benthic (seabed) zones (c, f), which included the nerito‐benthic (f, green), the shelf‐edge (f, 200–2,000 m; magenta), and deep‐sea (f, >2,000 m, blue) zones. Panels on the left (a, b, c) show mapping of the pseudo‐species richness, and panels on the right (d, e, f) represent latitudinal gradients in pseudo‐species richness. Each value in d, e, and f is the median of all longitudes for a given latitude. The vertical dashed line denotes the equator

**FIGURE 3:**
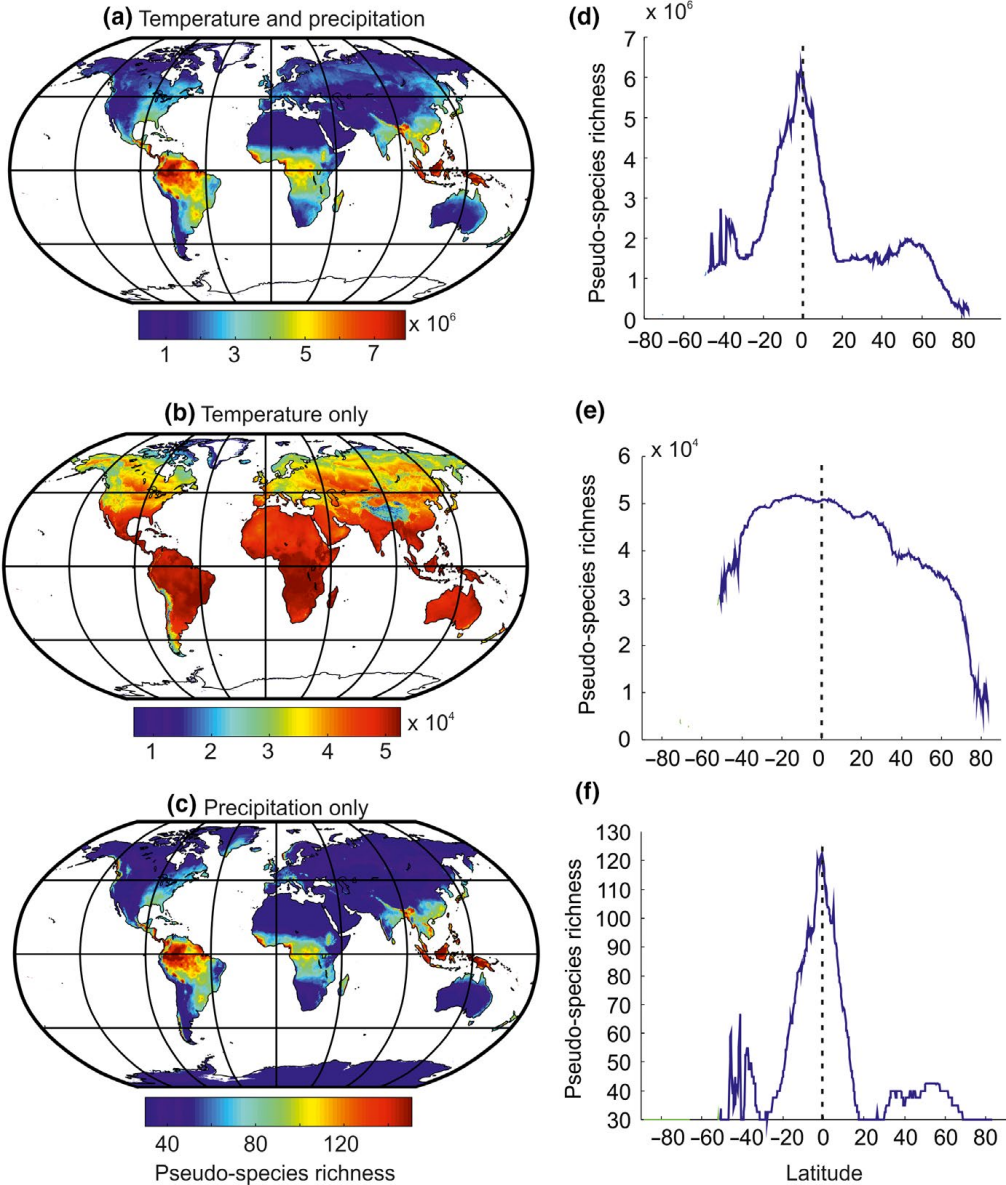
Terrestrial patterns in pseudo‐species richness based on (a, d) temperature and precipitation (b, e) temperature, and (c, f) precipitation only. Panels on the left (a, b, c) show mapping of the pseudo‐species richness, and panels on the right (d, e, f) represent latitudinal gradients in pseudo‐species richness. Each value in d, e, and f is the median of all longitudes for a given latitude. The vertical dashed line denotes the equator

We subsequently mapped the pseudo‐species richness by averaging monthly pseudo‐species richness for each domain (terrestrial vs. marine) and each marine zone (Figures 2 and 3). Our simulations were tested against field data at a global scale both by using information directly from the geographical cells (Figures 2 and 3) and also by looking at expected and observed LBGs (Figures 2 and 3 and Figure S1). Observed and predicted LBGs were obtained by calculating the median value of all longitudes, with a minimum of five values to estimate pseudo‐species and observed species richness median values.

**FIGURE 4:**
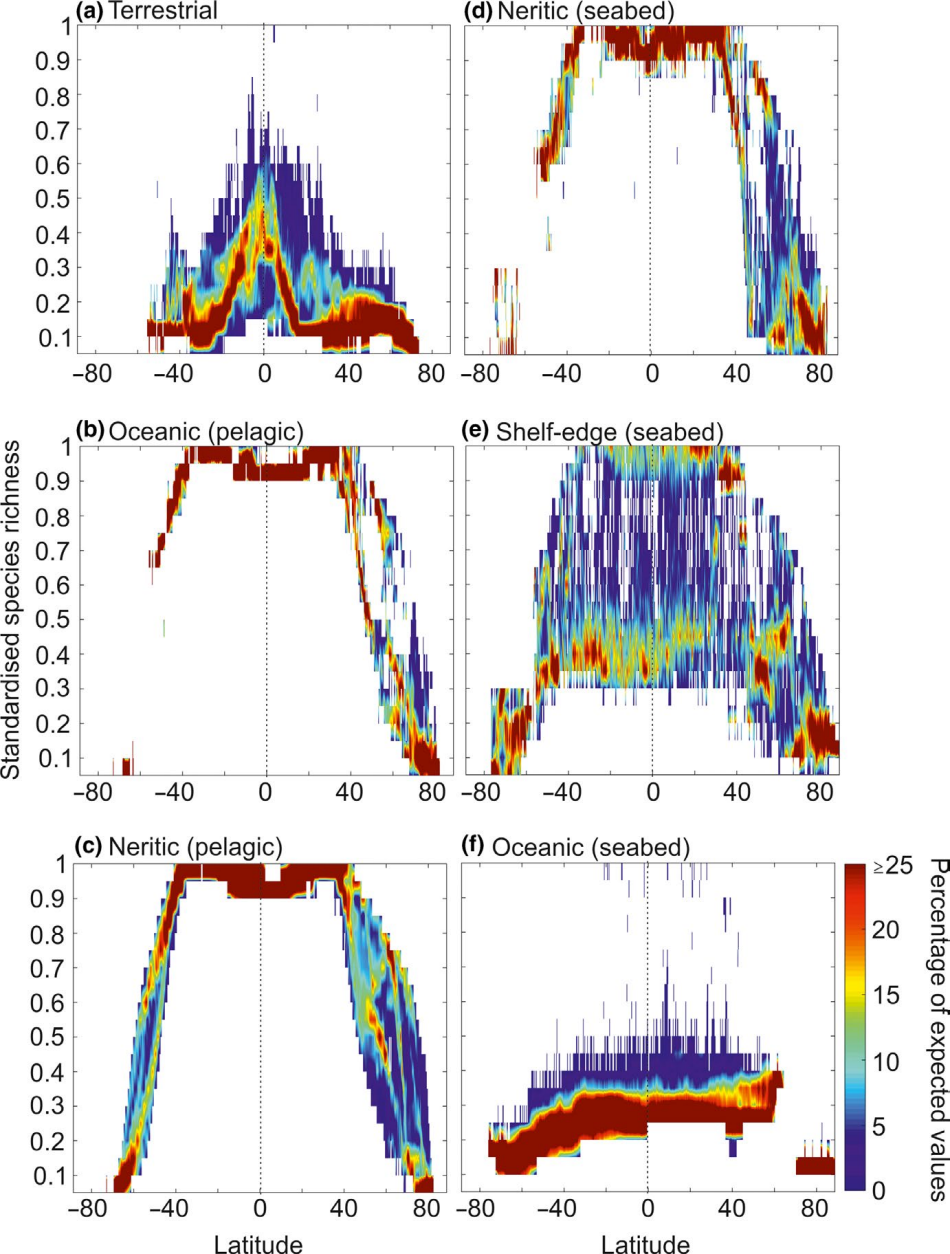
Modeled latitudinal biodiversity gradients (LBG) at all longitudes expressed as percentage of expected values. (a) Terrestrial LBGs. (b) Oceanic epipelagic LBGs. (c) Nerito‐pelagic zone. (d) Nerito‐benthic zone. (e) Shelf‐edge (2,000–200 m) zone. (f) Oceanic (seabed, i.e., >2,000 m) zone

We also calculated LBGs for all longitudes to examine how our perception of the LBG was influenced among longitudes. To do so, we standardized the pseudo‐species richness between 0 and 1 and estimated the number of times a given value of pseudo‐species richness was observed between 0 and 1, by a step of 0.05 for each latitude (Figure 4).

Because our goal was to model spatial biodiversity patterns rather than the exact number of species inside a taxonomic group, the number of species expected by the model could not be compared to the number of species within a taxonomic group. Therefore, we did not use tests commonly applied to examine both the similarity between observed and modeled species richness (e.g., the Kolmogorov–Smirnov test or the examination of the regression coefficient from ordinary least square regression; Rangel, Diniz‐Filho, & Colwell, 2007), but we used the Pearson correlation coefficient (Table 2, Table S1). To account for spatial autocorrelation in the geographical pattern of species richness (two dimensions), the degrees of freedom were recalculated to indicate the minimum number of samples (n*) needed to maintain a significant relationship at *p* = .05 (Beaugrand, Edwards, Brander, Luczak, & Ibañez, 2008; Helaouët, Beaugrand, & Reid, 2011; Rombouts et al., 2009). The smaller the n*, the less likely is the effect of spatial autocorrelation on the probability of significance. We preferred this technique to others (e.g., technique based on the calculation of the Moran's index or classical semivariograms) based on the assumption of isotropy, which is often violated as shown on the diversity of North Atlantic calanoid copepods by using (local) point cumulative semivariograms (Beaugrand & Ibañez, 2002).

#### 3.3.4 Step 4: Relationships with atmospheric processes

**FIGURE 5:**
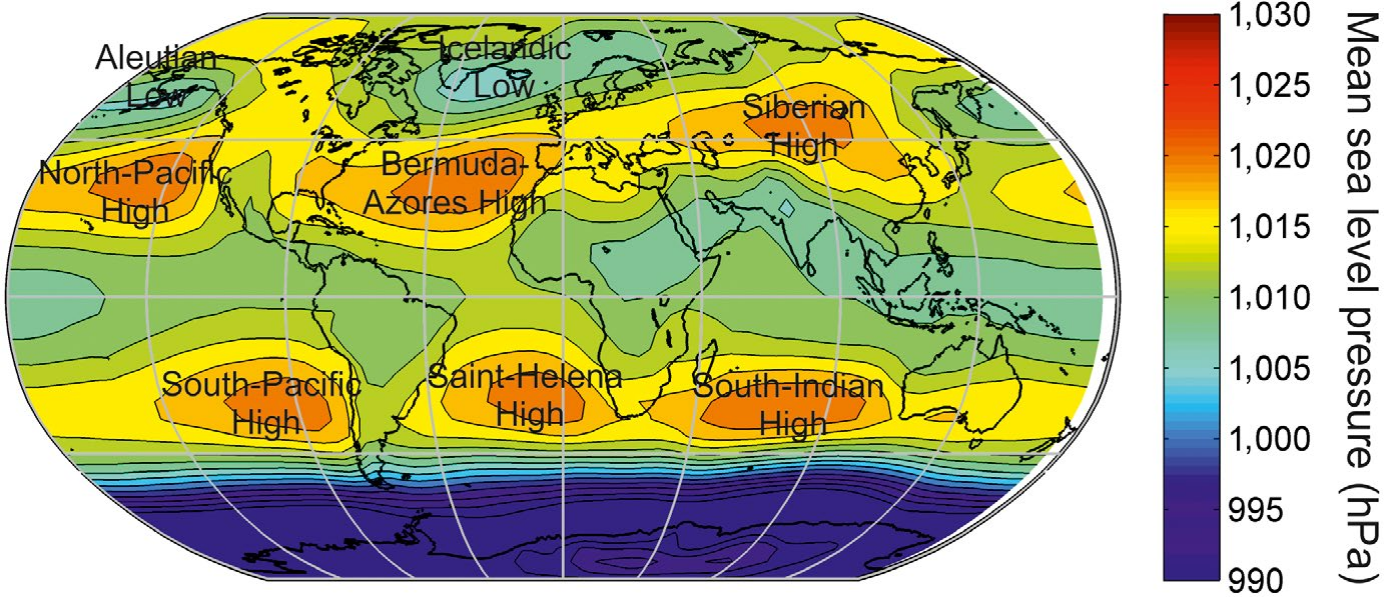
Global‐scale patterns in mean sea‐level pressure. The name of the semipermanent Highs and Lows is superimposed

To understand the origin of these patterns, we mapped averaged sea‐level pressure (SLP; Figure 5) and assessed the latitudinal clines in SLP, downward solar radiation at surface, and total precipitation over continents and the marine realm by calculating the median value of all longitudes for each latitude. A minimum of five values was needed to create an estimate for any given latitude (Figure S2). The same procedure was used for bathymetry in (a) the continental shelf (0–200 m), (b) the shelf‐edge (200–2,000 m), and (c) the ocean (>2,000 m; Figure S3). SLP and downward solar radiation at surface, known to affect biodiversity through temperature and precipitation, were not implemented into the model because they do not affect biodiversity directly.

#### 3.3.5 Step 5: Estimation of total biodiversity for each domain and zone (simulations at a 2° × 2° spatial resolution)

Even if similar environmental conditions (here, temperature and/or precipitation) occur in different oceanic and terrestrial regions, different species may be present according to Buffon's Law, which is also known as the first principle of biogeography (Lomolino et al., 2006). By designing a specific algorithm, we therefore enabled pseudo‐species having the same niche to be differentiated when they were permanently separated spatially on a monthly basis. We remind here that we did not consider year‐to‐year and longer time scale variability that clearly affects allopatric speciation (Rangel et al., 2018); this assumption is unlikely to alter global patterns of biodiversity or comparisons of biodiversity among realms at the time scale of our study. In practice, when an area with a contiguous presence was separated by at least one geographical cell (spatial grid 2° × 2°) from another contiguous area, the two areas were considered as occupied by two different species having the same thermal niche. Figure S4 shows the results of the application of our algorithm for different types of niche with each color representing a different species in each map. In our example, the same niche can create up to six pseudo‐species in the epipelagic zone (Figure S4). Note that the algorithm was only used at the 2° × 2° spatial resolution to reduce the computational time. Working at this resolution allowed us to estimate the mean number of pseudo‐species per niche without altering the spatial pattern in pseudo‐species richness.

We therefore performed the 12 further simulations at a 2° latitude × 2° longitude spatial resolution to estimate total pseudo‐species richness per domain and zone (Table 1, Figure 1). On land, five simulations (simulations 2, 3, 5, 7, and 8 in Table 1) were carried out to estimate total pseudo‐species richness (Figure 1 and Table 1). We used 1% of the niches when simulations were based on temperature and precipitation (total number of niches: 94,299,210 or 7,300,584 and therefore 942,992 or 73,005 niches following simulations, Table 1), all niches when they were precipitation‐based (930 or 72 niches, depending on the simulations, Table 1), and 25% of the niches (total number of niches: 101,397 so 25,349 niches) when temperature‐based. In the ocean, we performed a total of seven simulations (simulations 10, 12, 14, 16, 19, 21, and 23 in Table 1). We performed our simulations using only 25% of thermal niches (total number of niches: 101,397 so 25,349 niches; randomly selected).

The identification of several pseudo‐species per niche at a spatial resolution of 2° × 2°—in comparison with the 0.25° × 0.25° spatial resolution—only affects the total number of species we assessed for each realm and zone, but did not affect locally biodiversity. As for simulations performed at a 0.25° × 0.25° spatial resolution, monthly estimates in pseudorichness biodiversity were averaged annually and we retained the total number of pseudo‐species biodiversity.

#### 3.3.6 Step 6: Estimation of key biological parameters for understanding life organization

Some niches were incompatible with monthly environmental fluctuations and so not all niches from our pools (ψ_1_ in Table 3 and Table S2) were filled by a pseudo‐species. We therefore retained the number of niches for which at least one pseudo‐species occurs (ψ_2_ in Table 3 and Table S2). The ratio ψ_3 _= ψ_2_/ψ_1_ gives the percentage of niches that can be found in a given domain or ecological zone (Table 3 and Table S2). We assessed the mean number of pseudo‐species per niche for each domain or zone (ψ_4_ in Table 3 and Table S2). The total number of pseudo‐species ψ_5_ was assessed as follows:

(11) $$\psi_{5}=\psi_{1}\cdot \psi_{4}\cdot \phi$$

With ϕ = 100 when niches were based on temperature and precipitation (simulations were based on only 1% of the niches, Table 3 and Table S2), ϕ = 1 when niches were precipitation‐based (simulations were based on 100% of the niches, Table 3 and Table S2), and ϕ = 4 when niches were temperature‐based (simulations were based on only 25% of the niches, Table 3 and Table S2).

**TABLE 3:** Comparison of total pseudo‐species richness between domains and ecological zones

	Domain or zone (million km^2^)	Variable	Pool of niches (ψ_1_)	ψ_2_	ψ_3_	ψ_4_	ψ_5_ × 10^6^	ψ_6_ (ψ_7_–ψ_8_) × 10^4^	ψ_9_	ψ_10_
Land	Surface global (146.52)	T & P (1%)	942,992	527,442	55.93	20.33	1,072.5	2.3 (0.47–61.7)	0.02	35.93
	Surface global (146.52)	P (100%)	930	725	77.95	22.39	0.01	1.5 (0.43–60.3)	0.01	75.00
	Surface global (146.52)	T (25%)	25,349	25,164	99.27	38.04	3.82	241.0 (168.6–319.6)	1.64	45.35
Marine	Surface Global (355.44)	T (25%)	25,349	24,112	95.12	13.17	1.27	1,472.9 (781.7–2,058.7)	4.14	79.91
	Surface neritic (<200 m) (19.91)	T (25%)	25,349	24,112	95.12	53.11	5.12	15.7 (12.1–22.1)	0.78	42.61
	Surface oceanic (>200 m) (337.07)	T (25%)	25,349	23,903	94.29	16.11	1.54	1,164.1 (612.6–1,623.2)	3.45	80.55
	Benthic global (369.93)	T (25%)	25,349	24,446	96.43	83.17	8.13	18.8 (11.4–37.3)	0.05	98.63
	Benthic neritic (<200 m) (28.46)	T (25%)	25,349	24,446	96.43	56.42	5.51	12.7 (8.8–18.4)	0.44	69.31
	Benthic oceanic (>2,000 m) (301.84)	T (25%)	25,349	22,747	89.73	26.93	2.45	6.0 (1.4–18.8)	0.02	99.42
	Benthic shelf‐edge (200−2,000 m) (35.99)	T (25%)	25,349	23,600	93.10	90.65	8.5	7.5 (3.9–16.2)	0.21	94.55

Note

ψ_1_: pool of niches, ψ_2_: number of niches that can potentially be present in a domain or an ecological zone, ψ_3_: percentage of niches that can potentially be present in a domain or an ecological zone, PS: pseudo‐species, ψ_4_: mean number of pseudo‐species per niche, ψ_5_: total number of pseudo‐species, ψ_6,_ ψ_7,_ ψ_8_: median (ψ_6_), and first (ψ_7_) and third (ψ_8_) quartiles of the area (km^2^) occupied by a pseudo‐species, ψ_9_: percentage of the total area occupied by a pseudo‐species, ψ_10_: seasonal stability in pseudo‐species richness, T: temperature, P: precipitation.

We assessed the median area (ψ_6_ in Table 3 and Table S2), the first quartile (ψ_7_ in Table 3 and Table S2), and third (ψ_8_ in Table 3 and Table S2) quartile covered per pseudo‐species in each domain and ecological zone. Area (km^2^) occupied by a pseudo‐species was calculated as follows (Beaugrand & Ibañez, 2002):

(12) $$d\left(i,j\right)=6,377.221\times h_{i,j}$$

with *d_i,j_* being the geographical distance between point *i* and *j*, the constant the Earth radius and *h_i,j_* computed as follows (Beaugrand & Ibañez, 2002):

(13) $$h_{i,j}=\text{arcos}\left(\sin\gamma_{i}\sin\gamma_{j}+\cos\gamma_{i}\cos\gamma_{j}\cos g\right)$$

With ϒ*_i_* the latitude (in radians) at point *i*, ϒ*_j_* the latitude (in radians) at point *j*, and *g* the difference in longitude between *i* and *j*.

The percentage of the total area occupied by a single pseudo‐species was given by ψ_9_ (Table 3 and Table S2)_._ An index of monthly stability in pseudo‐species richness ψ_10_ was assessed for each geographical cell as follows:

(14) $$\psi_{10}=\frac{\sum_{i=1}^{m}\varphi_{i}}{m\Phi }$$

With *m* = 12 months, *φ* is monthly pseudo‐species richness and Ф total pseudo‐species richness for a given geographical cell. When *ψ*_10_ tends toward 1, monthly stability was high. When it tends toward 0, monthly stability was weak.

#### 3.3.7 Step 7: Scaling of marine and terrestrial total pseudo‐biodiversity to current estimates of biodiversity

To investigate whether our model could reproduce the difference in total species richness observed among realms, we scaled total pseudo‐biodiversity of the marine and terrestrial realms by using information on catalogued and estimated eukaryotic biodiversity from Mora and colleagues (Mora et al., 2011). As previously stated, we focused on eukaryotic biodiversity because METAL has only been tested on eukaryotes so far. Mora and coworkers (Mora et al., 2011) reported 1,427,256 catalogued species with 1,233,500 terrestrial and 193,756 marine species. They also estimated the total number of eukaryotes to be 10,950,000 with 8,740,000 terrestrial and 2,210,000 marine species (Mora et al., 2011).

For this analysis, we considered the total estimation of terrestrial pseudo‐biodiversity based on precipitation and thermal niches (line 2 in Table 3 and Table S2; we called this number Θ^T^) and the estimation of marine pseudo‐biodiversity based only on temperature (lines 6, 7, Table 3 and Table S2). For the marine realm, we considered the nerito‐ and the holo‐pelagic ecological zone (lines 6 and 7 in Table 3 and Table S2) as well as the nerito‐benthic, the shelf‐edge (200–2,000 m), and oceanic seabed (>2,000 m; lines 9, 10, and 11 in Table 3 and Table S2). We summed total pseudo‐biodiversity of all marine ecological zones (hereafter Θ^M^). Earth pseudo‐biodiversity Θ was therefore assessed, as follows:

(15) $$\Theta =\Theta^{T}+\Theta^{M}$$

To convert total pseudo‐biodiversity (Θ) into total biodiversity, we divided Equation (15) by either the total number of catalogued (1,427,256) or estimated (10,950,000) species. We performed this analysis with two runs: the first being based on 930 precipitation niches (Table 3) and the second on 72 precipitation niches (Table S2).

## 4 RESULTS AND DISCUSSION

### 4.1 Global biodiversity patterns

We filled the land and sea of an empty planet with biodiversity using models from the METAL theory (Text S1, Table 1). Our models were based on temperature for the ocean and both temperature and precipitation for land because water is essential to explain terrestrial biogeographic patterns (Sunday, Bates, & Dulvy, 2012; Whittaker, 1975). We did not include edaphic (e.g., pH, soil), sediment (e.g., sediment size and type), other ecological dimensions (e.g., oxygen and nutrients), or human disturbances that may also influence regional biodiversity (Text S3). Finally, because marine habitats are more vertically structured, we split the ocean into five zones: nerito‐pelagic, holo‐pelagic, nerito‐benthic, shelf‐edge, and the deep seabed (glossary, Text S2).

On land, our model predicted high values of terrestrial biodiversity over Indonesia, Malaysia, New Guinea, the Philippines, Central America, and Africa (Figure 2a) in agreement with reported studies (Cox & Moore, 2000; Lomolino et al., 2006; Myers, Mittermeier, Mittermeier, da Fonseca, & Kent, 2000). Locally, biodiversity was high in the northeastern part of Madagascar, Indo‐Burma, and Tropical Andes hotspots (Myers et al., 2000; Rangel et al., 2018). Globally, biodiversity on land was twofold greater than in the sea (Figure 2a–c). Reconstructed biodiversity patterns were close to observed patterns in nature (Table 2); correlations ranged between 0.66 and 0.79 for a variety of taxonomic groups from plants to mammals. Species richness reconstructions on land performed better when based on both temperature and precipitation together, with the exception of reptiles (especially lizards and snakes) for which the unique use of temperature reproduced their biodiversity well (Figure 3 and Table S1).

In agreement with other biogeographic studies (Kaschner, Tittensor, Ready, Gerrodette, & Worm, 2011; Rombouts et al., 2009; Tittensor et al., 2010), large‐scale pelagic biodiversity patterns in the sea were more uniform than on land and high biodiversity was observed at midlatitudes in contrast to the equator for land (Figure 2b). Correlations between predicted and observed biodiversity patterns were 0.71–0.89 for the epipelagic (nerito‐pelagic and holo‐pelagic, Text S2) zone (e.g., cetacean and foraminifera), 0.64–0.71 in the nerito‐benthic zone (where light reaches the seabed; e.g., coral and seagrass), and 0.62–0.84 for the neritic zone (nerito‐pelagic and nerito‐benthic; e.g., squid and non‐oceanic sharks). Although our model was only tested in the neritic realm (0–200 m, Table 2) and the holo‐pelagic zone because sampling is scarce in the deep ocean (Danovaro, 2012; Watling, Guinotte, Clark, & Smith, 2013), we consider that it can also inform global‐scale biodiversity patterns in the deep sea (Figure 2c). Modeled benthic biodiversity was higher over shallow regions and much lower over deep regions. It was also high over many coastal regions of the Indo‐Pacific, the Red Sea, shallow regions of the Gulf of Mexico, the Mediterranean Sea, and to a lesser extent the southwestern part of Europe. In the deep sea, modeled benthic biodiversity was higher over the mid‐ocean ridge and seamounts, a prediction confirmed by observations (Kelly, Shea, Metaxas, Haedrich, & Auster, 2010; Morato, Hoyle, Allain, & Nicol, 2010).

Dispersal, classically defined as the movement of individuals away from a source population, varies among taxonomic groups and species within a taxonomic group (Beaugrand, 2015a; Lidicker & Stenseth, 1992; Palumbi, 1992). Because we did not make any specific simulations for a taxonomic group here (i.e., dispersal was assumed to be identical among taxonomic groups), it follows that correlations between modeled and observed global biodiversity patterns may have been affected. Correlations were surprisingly similar among taxonomic groups, however (Table 2), and were slightly higher for groups of the marine epipelagic realm where dispersal is typically large (Palumbi, 1992). Some terrestrial reptiles had smaller correlations, which may be explained by a smaller dispersal capability and their ecology (Todd, Willson, & Gibbons, 2010).

The predicted LBGs were distinct among realms (Figure 2d–f and Figure S1). Although a peak of biodiversity was predicted between the tropics on land, with a maximum at the equator (Figure 2d and Figure S1a), this was not so in the surface ocean (epipelagic zone, 0–200 m) where a maximum occurred over subtropical regions with a reduction in the tropics and a slight equatorial increase (Figure 2e–f and Figure S1a–b). The predicted terrestrial and marine LBGs were highly correlated with observed LBGs (Cox & Moore, 2000; Economo, Narula, Friedman, Weiser, & Guénard, 2018; Lomolino et al., 2006; Rombouts et al., 2009; Tittensor et al., 2010; Figure S1 and Table 2).

While benthic biodiversity exhibited similar latitudinal patterns to pelagic biodiversity in shallow regions, closer examination showed a slight reduction, rather than an increase, at the equator (Figure 2e–f). Biodiversity was low throughout deep‐sea areas with a noticeable decline above 60°N (Figure 2f). Shelf‐edge biodiversity was higher in Northern than in the Southern Hemisphere (SH) (40°S–40°N) due to lower average SH bathymetry (Figure S3). Although LBGs have been extensively documented (Cox & Moore, 2000; Economo et al., 2018; Lomolino et al., 2006; Rombouts et al., 2009; Tittensor et al., 2010), no theory has been proposed to explain the different LBGs on land and in the sea within a unifying framework before.

We also calculated the LBGs for each longitude to examine the influence of longitudes on our perception of the LBGs (Figure 4). Although for some realms the influence was minor, this was not so for the benthic realm, especially the shelf‐edge and the nerito‐benthic realms. Intermediate patterns were observed over the shelf‐edge (2,000–200 m; Figures 1f and 4). Depending upon bathymetry, the shelf‐edge exhibited LBGs typical of shallow or deep regions (Figure 4). This analysis explained why high latitudinal variability was observed under some circumstances (Figure S1d).

We suggest that the different terrestrial and marine LBGs are caused by water limitation in the subtropics due to high‐pressure cells limiting precipitation (Figure 5). These cells cover a more limited area in the Southern Hemisphere, which explains why terrestrial biodiversity was slightly higher (Figure 2a,d). While high‐pressure cells limit terrestrial biodiversity because of their negative influence on precipitation (Figure S2), it is the place where pelagic biodiversity is highest because temperature is the only climatic factor (Figure 2b,e).

Our unifying framework therefore explains why biodiversity peaks at the equator on land, why it peaks at midlatitudes in the epipelagic ocean, and why it is expected to remain high over neritic (pelagic and benthic) regions between tropics. In addition, it suggests that deep‐sea biodiversity should be little affected by latitudes between 40°S and 40°N. We propose that a simple principle, a mathematical constraint on the number of species that can coexist locally, arising from the niche–environment (here climate–environment) interaction, is at the origin of LBGs observed among realms. We have previously called this constraint the chessboard of life (Beaugrand et al., 2018). The rate of net diversification is important because it affects the degree of niche occupancy in a given area. We have shown previously that niche saturation (i.e., the number of occupied niches in an area) was higher in the tropics than in temperate systems, probably because of greater net tropical diversification rates (Dowle, Morgan‐Richards, & Trewick, 2013; Jablonski, Roy, & Valentine, 2006) or faster species turnover in extratropical regions (Weir & Schluter, 2007). However, we have also shown that polar systems had the highest degree of niche saturation because the number of niches in polar systems was much lower (Beaugrand et al., 2018). Our results therefore suggest that while speciation is fundamental to fill the chessboard of life, this is not what determines large‐scale biodiversity patterns. The arrangement of biodiversity may primarily result from a mathematical constraint that originates from a fundamental interaction: the niche–environment interaction.

### 4.2 Total biodiversity comparisons among realms

Modeled total biodiversity was also estimated for each realm and ecological zone at a coarser spatial resolution (Table 3). Spatial patterns in pseudo‐species richness based on 0.25° × 0.25° and 2° × 2° were highly correlated (*r* = .99, *p* < .05, *n* = 15,929, n*=1), indicating patterns were very close. We first assumed that a niche led to the establishment of only one pseudo‐species. With two climatic dimensions, the terrestrial domain had greater total pseudo‐biodiversity than the marine domain (94 for the terrestrial vs. 0.1 million pseudo‐species). Of the 942,992 niches we used (**ψ_1_** in Table 3), 55.93% of the niches led to the establishment of a pseudo‐species in the terrestrial domain while between 93.1% and 96.4% of the marine niches (25% of the pool of niches, 25,349) gave a pseudo‐species (**ψ_2_** and **ψ_3_** in Table 3). The higher number of terrestrial niches/pseudo‐species was caused by the addition of a second climatic dimension.

Next, we considered that a niche could lead to the establishment of several pseudo‐species provided they were separated spatially from each other (Buffon's Law; Lomolino et al., 2006); this analysis aimed to reveal the potential influence of allopatric speciation on biodiversity. On average, a terrestrial niche gave 20.3 pseudo‐species when temperature and precipitation were considered, and a marine niche led to between 13.1 and 90.6 pseudo‐species at the surface and the shelf‐edge, respectively (**ψ_4,_** Table 3). Multiplying the number of niches (**ψ_4_**) by the mean number of pseudo‐species per niche (**ψ_1_**) led to the number of pseudo‐species expected for each domain or zone (**ψ_5_**). The greater number of potential terrestrial niches created higher total pseudo‐biodiversity (1,072.5 million terrestrial vs. 23.1 million marine pseudo‐species; Aarssen, 1997). The spatial homogeneity of the epipelagic zone means there is less potential for allopatric speciation than in the seabed (**ψ_4_**, Table 3), which explains why there are more benthic pseudo‐species (**ψ_5_** = 1.3 surface vs. **ψ_5_** = 8.13 million benthic pseudo‐species). Similarly, more speciation is likely in the neritic zone, which explains the higher pseudo‐biodiversity (Tittensor et al., 2010). The model also predicts the shelf‐edge should have a higher total biodiversity than the nerito‐benthic zone. Although the shelf‐edge zone has been less investigated, a unimodal biodiversity pattern with depth has been suggested with biodiversity peaking between 1,000 m and 3,000 m (Rex, 1981). Because the number of niches was approximately similar among all marine zones (**ψ_2_**), it was the potential for allopatric speciation (**ψ_4_**) and the area of a realm that most influenced total marine biodiversity (**ψ_5_**, Table 3).

High biodiversity is associated with ecosystem stability (Duffy, 2002). However, this should not confer more resistance/resilience to environmental changes in the terrestrial domain (even though terrestrial total pseudo‐biodiversity was higher than marine) because, in our model, the mean spatial range occupied by a terrestrial pseudo‐species was lower (**ψ_6–8_** in Table 3); many studies have suggested that species resistance is a function of the area occupied by a species (MacArthur & Wilson, 1967; Thomas et al., 2004). The same also applies for marine zones with a higher total pseudo‐biodiversity, for example, neritic and shelf‐edge zones. In terms of percentage area terrestrial pseudo‐species covered the same median area as shelf‐edge species (**ψ_9_**, Table 3).

Our simulations suggest that spatial heterogeneity increases local biodiversity by enabling the coexistence of more niches and by promoting allopatric speciation (Figure 1 and Table 3). Similarly, monthly stability in pseudo‐biodiversity (**ψ_10_**, Table 3) was correlated negatively with total pseudo‐biodiversity (*r* = −.67, *p* = .06, *n* = 6, log‐transformed variables), which suggests that higher temporal heterogeneity promotes higher biodiversity by enabling more species turnover. The nerito‐pelagic zone was characterized by low monthly stability (Table 3), which was due exclusively to temperature. Precipitation, however, was the main cause of terrestrial temporal heterogeneity (Table 3). The deep benthic zone was highly stable.

We scaled pseudo‐biodiversity to both catalogued (1,233,500 terrestrial and 193,756 marine species) and estimated (8,740,000 terrestrial and 2,210,000 marine species) eukaryotic biodiversity (Mora et al., 2011). We implemented the model twice: firstly for 930 precipitation niches and secondly for 72 precipitation niches. Decreasing the number of precipitation niches reduces model accuracy because having fewer niches provides more stepwise transitions but large‐scale biodiversity patterns were highly correlated (*r* = .92, *p* < .05, *n* = 15,929, n*=3), with similar conclusions in terms of niches, biodiversity, and stability (Table 3 vs. Table S2). By decreasing the number of niches, the simulation better approaches nature. With 930 precipitation niches, total terrestrial pseudo‐biodiversity—scaled to both catalogued and estimated eukaryotic species—gave 1,397,047 (10,718,238) terrestrial and 30,208 catalogued (231,762 estimated) marine species. Therefore, while this simulation predicted that biodiversity should be higher in the terrestrial than the marine domain, it underestimated catalogued biodiversity by factor of 6.4 (catalogued) and 9.53 (estimated), respectively. When precipitation niches were reduced however (*n* = 72), total pseudo‐biodiversity scaled to catalogued (estimated) species gave 1,111,186 (8,825,091) for the terrestrial domain and 316,069 (2,242,908) for the marine domain. Our model therefore reproduced the difference in observed or estimated biodiversity between the marine and terrestrial domains well, although results depended upon the number of selected precipitation niches. Interestingly, our estimate of the deep‐sea benthic biodiversity (894,881 benthic species in areas below 2,000 m and 256,278 in areas between 2,000 m and 200 m) is close to what has been calculated in previous studies (Grassle & Maciolek, 1992; Snelgrove, 1999). Species density is expected to be higher over shelf‐edge (200–2,000 m) than deep sea (ψ_2‐4_ in Table S2) but because the latter realm is larger (301 vs. 36 million km^2^, Table S2), there are more total number of species in the deep‐sea benthic realm.

In our model, we assumed that dispersal of each pseudo‐species was high enough to fully occupy a given spatial range (i.e., a contiguous area where environmental conditions are suitable for a pseudo‐species). In other words, biodiversity patterns were based on the assumption of full distributional range occupancy reached at equilibrium. When the potential for allopatric speciation was considered, the existence of a single barrier to dispersal (i.e., a space with unsuitable environmental conditions in term of temperature or precipitation, or both) was sufficient enough to prevent a species to also occur in another region with suitable environmental conditions, and thereby, another species colonized the area. Because species disperse farther in the oceanic than in the terrestrial realm (Kinlan & Gaines, 2003; Palumbi, 1992), this assumption may have inflated marine biodiversity estimates (and especially seabed biodiversity estimates, see ψ_4 _in Table 3) and therefore diminished the contrast of total biodiversity between the terrestrial and the marine realms.

Our model did not consider the implications of past climate change to estimate the potential for allopatric speciation. Although this will have no effect on large‐scale biodiversity patterns, this may have influenced our estimations of total biodiversity for each realm. This influence would be consistent among realms, however. Consideration of past climate change would reduce the mean number of species per niche in all realms. However, the effect is likely to be more prominent in the terrestrial and in the marine neritic (benthic and pelagic) realms, less important for the shelf‐edge realm, and small for the deep‐sea benthic realms.

### 4.3 Better understanding of processes influencing biodiversity

Factors that contribute to the biodiversity are numerous and belong to a large range of temporal and spatial scales (Lomolino et al., 2006). Many authors have made significant attempts to identify the primary factor involved in global biodiversity patterns, and a large number of explanations have been proposed (Allen, Brown, & Gillooly, 2002; Beaugrand et al., 2013; Cardillo, Orme, & Owens, 2005; Colwell & Lees, 2000; Connell & Orias, 1964; Darlington, 1957; Gillooly, Allen, West, & Brown, 2005; Hawkins et al., 2003; Hubbell, 2001; MacAthur, 1965; O'Brien, Field, & Whittaker, 2000; Rohde, 1992; Rosenzweig, 1995; Turner & Hawkins, 2004). Some authors have proposed null or neutral models such as the neutral model of biodiversity and biogeography (Hubbell, 2001) and the mid‐domain effect (Colwell & Hurtt, 1994). Others have suggested that LBGs may originate from the larger area of the tropical belts (Rosenzweig, 1995). Evolutionary explanations have also been put forward (Mittelbach et al., 2007). Perhaps the most compelling hypotheses have been those that invoke an environmental control of biodiversity such as environmental stability or energy availability (Beaugrand, Reid, Ibañez, Lindley, & Edwards, 2002; Rutherford, D'Hondt, & Prell, 1999; Tittensor et al., 2010). Although temperature (both terrestrial and marine realms) and water availability such as precipitation (terrestrial realm) have been often suggested to explain large‐scale patterns in the distribution of species (Beaugrand et al., 2010; Lomolino et al., 2006; Rangel et al., 2018; Tittensor et al., 2010), mechanisms by which those parameters control LBGs have remained elusive. More recent findings have suggested an important influence of species’ niche in the generation of patterns of biodiversity (Beaugrand et al., 2013, 2015, 2018; Beaugrand & Kirby, 2018b; Hawkins et al., 2003; Rangel et al., 2018).

Here, we suggest that biodiversity is mathematically constrained by an underlying structure we have previously called the chessboard of life (Beaugrand et al., 2018), which fixes the maximum number of species that can coexist regionally and controls global‐scale biodiversity patterns. Although there are both a large part of contingency in biodiversity and species’ occurrence depends upon local stochastic processes (Hubbell, 2001), nature appears ordered and intelligible at a global scale.

We suggest that LBGs are different in the marine and terrestrial realms because of the existence of a second important dimension in the climatic niche of terrestrial species: water availability. (This parameter was estimated in this paper using monthly precipitation.) Although temperature is a key factor in the marine realm (Beaugrand et al., 2010; Rombouts et al., 2009; Tittensor et al., 2010), both temperature and precipitation are needed in the terrestrial realm (Hawkins et al., 2003; Rangel et al., 2018; Whittaker, 1975).

The differential influence of high sea‐level pressure cells on climate explains the strong difference observed between LBGs in the terrestrial and marine realms. While high sea‐level pressure cells influence positively marine biodiversity through the effect of temperature (mean and temporal variability), they affect negatively terrestrial biodiversity through its adverse effects on precipitation (Figure 5 and Figure S2). Identification of the root mechanisms that explain both LBGs is important because it provides a clue on the primary cause of large‐scale biodiversity patterns. High biodiversity can only be observed where the number of niches is high. More niches can be created at the middle part of climatic gradient (either temperature or precipitation). Niche packing, also known as the niche‐assembly or the structural theory (MacAthur, 1965; Pellissier, Barnagaud, Kissling, Sekercioglu, & Svenning, 2018; Turner & Hawkins, 2004), resulted here from a mid‐domain effect (Colwell & Lees, 2000) in the Euclidean space of the climatic niche (Beaugrand et al., 2013). The number of niches, and thereby the number of species, deeply decreases in areas characterized by extremely low precipitation (Figure S2) and to a lesser degree higher temperature (Figure 3). In the marine realm, the equatorial decrease in biodiversity is due to too high temperature at the equator; see Figure S2 in Beaugrand and colleagues (Beaugrand et al., 2013).

The importance of the second dimension of the climatic niche of terrestrial species (i.e., precipitation) also explains why there are more terrestrial than marine species (Table 3): It increases substantially the number of niches (**ψ_1_**), diminishes the mean distributional range of a species (**ψ_6–8_**), and leads to an increase in potential allopatric speciation (**ψ_4_**). As a result, terrestrial species have a smaller mean spatial range than marine species (**ψ_6‐8_**) and the influence of allopatric speciation is probably more pronounced (**ψ_4_**), exacerbating the contrast between marine and terrestrial biodiversity (**ψ_5_**). We have seen previously that our estimations may be affected by dispersal. Because marine dispersal is high in the marine realm (Palumbi, 1992), our estimations of the number of pseudo‐species per niche may be too large, although they would reinforce our conclusion on the strong species biodiversity contrast between land and sea.

## 5 CONCLUSION

We therefore conclude by stating that a simple principle, a mathematical constraint on the number of species that can coexist locally, which originates from the niche–environment (here niche‐climate) interaction, is at the origin of LBGs and the biodiversity differences observed among realms. Climate has a primordial influence on biodiversity. Mean and spatial gradient in SLP influence both temperature and precipitation, which have a direct influence on species physiology. Interaction between those parameters and species’ climatic niche generates a mathematical constraint to the maximum number of species that can establish locally, what we called previously the chessboard of life. An additional climatic dimension in the terrestrial realm (i.e., precipitation), which multiplies the number of terrestrial niches, may explain why there are more species in this realm despite the fact that life first emerged in the sea. Spatial heterogeneity may increase biodiversity by allowing more niches to coexist and by increasing allopatric speciation. While speciation is fundamental because it creates species, this process is constrained by the maximum number of niches available locally.

## CONFLICT OF INTEREST

None declared.

## AUTHOR CONTRIBUTION

**Gregory Beaugrand:** Conceptualization (lead); Data curation (lead); Formal analysis (lead); Funding acquisition (lead); Investigation (lead); Methodology (lead); Project administration (lead); Resources (lead); Software (lead); Supervision (lead); Validation (lead); Visualization (lead); Writing‐original draft (lead); Writing‐review & editing (lead). **Richard Kirby:** Writing‐original draft (supporting); Writing‐review & editing (supporting). **Eric Goberville:** Data curation (supporting); Writing‐original draft (supporting); Writing‐review & editing (supporting).

## Supporting information

Supplementary Material

## Data Availability

All data originating from our model are available through a Web site http://metaltheory.weebly.com/
