# Supplementary material for: The mathematical influence on global patterns of biodiversity
Source: Ecol Evol. 2020 Jun 11;10(13):6494–511. doi: 10.1002/ece3.6385 (PMC7381758; doi:10.1002/ece3.6385)
Supplement: Supplementary file 1 — Supplementary Material [file ECE3-10-6494-s001.docx]

Supplementary Materials

**The mathematical control of global biodiversity**

Text S1-S3

Tables S1-S2

Figures S1-S4

References

**Text S1: Overview of the MacroEcological Theory on the Arrangement of Life**

We applied a framework based on the MacroEcological Theory on the Arrangement of Life (METAL)(Beaugrand, et al. 2014, Beaugrand 2015a, Beaugrand 2015b, Beaugrand, et al. 2015, Beaugrand and Kirby 2018a, Beaugrand, et al. 2018). The theory uses the concept of the ecological niche *sensu* Hutchinson (Hutchinson 1957) as a macroscopic elementary brick to understand how species fluctuate in time and space and how communities form and are altered by environmental changes, including climate change. Although Grinnell (Grinnell 1917), Elton (Elton 1927), Hutchinson (Hutchinson 1978), Whittaker (Whittaker 1975) and more recently Chase and Leibold (Chase and Leibold 2003) developed or used the concept of the niche, they did not explicitly connect this concept to the arrangement of life from the species to the community level. At the species level, it is well-known that the niche/environment interaction enables the species’ spatial distribution to be estimated with confidence at a macro-scale, thus explaining the strong development of Ecological Niche (ENM), Spatial Distribution (SDM) and macro-physiological Models (Albouy, et al. 2012, Araujo and Guisan 2006, Cheung, et al. 2008, Helaouët, et al. 2011, Lenoir, et al. 2011, Raybaud, et al. 2013). Indeed, Brown’s theory (Brown 1984) states that species’ local density and range are the result of the species’ ecological niche. Far less known, however, is the fact that phenology and local year-to-year changes in species abundance - and their interaction within the spatial range of a species - can also be inferred from the knowledge of the niche (Beaugrand, Mackas, et al. 2013, Beaugrand, Goberville, Luczak and Kirby 2014, Beaugrand and Kirby 2016). At the species level, METAL enables the understanding and prediction of annual abundance, phenology and biogeography (Beaugrand, Goberville, Luczak and Kirby 2014, Beaugrand 2015b, Beaugrand and Kirby 2018b). METAL has also been applied recently to explain the apparent inconsistency in the relationships between climate and species on a year-to-year basis (Beaugrand, Mackas and Goberville 2013, Beaugrand and Kirby 2016). Predictions of the effects of climate change on species are possible when the niche is assessed from physiology (fundamental niche) or spatial distribution (realised niche). METAL not only explains the spatial and temporal responses of species to climatic variability or climate change but it also enables these apparently independent responses to be connected in a unique theoretical framework (Beaugrand and Kirby 2018b).

At a higher organisational level, the METAL theory may be used to better understand how communities organise themselves and how they may be affected by environmental changes (Beaugrand and Kirby 2018a). Although the METAL theory has been tested for a limited number of species until now (Beaugrand, Mackas and Goberville 2013, Beaugrand, Goberville, Luczak and Kirby 2014, Beaugrand 2015b, Beaugrand and Kirby 2016) the available evidence available suggests that the ecological niche of each species influences their spatial and temporal responses to climate change. Under this hypothesis we can create a pool of pseudo-species that each has a unique niche after the principle of competitive exclusion of Gause (Gause 1934) while considering niche overlapping (Beaugrand, Rombouts, et al. 2013, Beaugrand, Edwards, Raybaud, Goberville and Kirby 2015). Species are allowed to colonise a given oceanic region so long as they can survive changes in the environmental regime at different temporal scales. By reconstructing pseudo-communities, we are able to investigate (i) the origin of various ecogeographic patterns (e.g. latitudinal gradients in biodiversity, Rapoport’s effect, Infrequency Law)(Beaugrand, Rombouts and Kirby 2013, Beaugrand 2015b), (ii) community processes such as seasonal succession, (iii) properties of communities and (iv) their consequences for ecosystem functioning, and both regulating and provisioning services (Beaugrand, Rombouts and Kirby 2013, Beaugrand 2015a, Beaugrand, Edwards, Raybaud, Goberville and Kirby 2015). The theory therefore explains how marine pelagic eukaryotes are arranged on Earth and proposes that biodiversity is influenced by climate and environment to a large extent. This influence mainly takes place through the interactions between the species ecological niche and both climatic and environmental changes. This interaction determines in large part the arrangement of life in the oceans at different organisational levels, from the species to the ecosystem level and from small to large ecosystems (Beaugrand 2015b).

Recently, we have theoretically investigated palaeo (mid-Pliocene and Last Glacial Maximum or LGM), contemporaneous (1960-2013) and future (2080-2100) changes in biodiversity in the context of global climate change to evaluate the sensitivity and vulnerability of biodiversity to climate change (Beaugrand, Edwards, Raybaud, Goberville and Kirby 2015). This theoretical work has revealed that climate change may rapidly alter marine biodiversity over large oceanic regions and that the intensity of this reorganisation will depend on the magnitude of warming. If global warming is small (RCP2.6) the study has shown that biological changes would reflect 25.3% of the amount of change observed between the mid-Pliocene and today, or 15.5% of the amount of change seen between the LGM and the present day; neither are that different to annual variability (1960-2013), and so it may be benign overall. If warming is moderate (RCP4.5) the changes in marine biodiversity will be three-times more extensive and at least twice as strong in magnitude than changes observed over the last 50 years. If global warming is severe (RCP6.0 and 8.5) we expect that between 50 and 70% of the global ocean will experience a change in marine biodiversity equivalent to, or higher than, that experienced between the LGM/mid-Pliocene and today.

**Text S2: Glossary (by alphabetic order)**

**Benthic zone:** region at the lowest level of the neritic (bathymetry of 0-200m) or the oceanic (bathymetry >200m) zone.

**Epipelagic zone:** zone of the ocean ranging from 0 to 200m.

**Holo-pelagic zone**: pelagic oceanic (i.e. bathymetry above 200m) zone.

**Pelagic zone**: the water column of the open sea in neritic and oceanic regions.

**Neritic zone**: continental shelf with a bathymetry between 0 and 200m.

**Nerito-benthic zone:** benthic zone of the continental shelf (i.e. region with a bathymetry between 0 and 200m).

**Nerito-pelagic zone:** pelagic zone above the continental shelf (i.e. region with a bathymetry between 0 and 200m).

**Oceanic zone**: regions with a bathymetry above 200m.

**Total pseudo-biodiversity**: total number of pseudo-species in a domain (marine or terrestrial) and an ecological zone (e.g. nerito-pelagic zone).

**Text S3: Limits of our framework**

The ecological niche is multidimensional. Unfortunately, and as recognised by Hutchinson (Hutchinson 1978), it is impossible to use all niche dimensions and so it is important to select a few that control a large part of the spatial distribution of species. The climate variability hypothesis states that the latitudinal range of species is primarily determined by their thermal tolerance (Stevens 1989). In the marine realm, temperature is a key variable because i) it is the result of many hydro-climatic processes (Beaugrand, et al. 2008) and ii) it exerts an effect on many fundamental biological and ecological processes (Sunday, et al. 2012). We therefore assumed that the main driver by which atmospheric forcing may affect biological communities is sea temperature because many studies showed that this parameter has a cardinal influence on species physiology, biology and ecology (Beaugrand, et al. 2002, Brown, et al. 2004, Pörtner and Farrell 2008) through its control of biological processes from the molecular to the cell and the organism levels (Brown, Gillooly, Allen, Savage and West 2004). Temperature therefore alters the growth, reproduction, mortality, and the behaviour of organisms at the species level and so it also affects biotic interactions (positive or negative) at the community level (Kirby and Beaugrand 2009, Schmidt-Nielsen 1990). At a global scale, temperature patterns largely determine the location of biogeographic provinces and biomes and so modulates ecological services such as food production and carbon sequestration (Beaugrand, et al. 2010, Sarmiento, et al. 2004). It is therefore not surprising that many biogeographical studies have revealed a cardinal influence of temperature on marine biodiversity (Rombouts, et al. 2009, Rombouts, et al. 2010, Rutherford, et al. 1999, Sunagawa, et al. 2015). To estimate biodiversity on land, the use of temperature and water availability was essential to model terrestrial species range (Roubicek, et al. 2010); in particular, these parameters are closely related to species, ecosystem and biome distribution (Bartlein, et al. 1986, Pearman, et al. 2008, Whittaker 1975).

However, we are aware that multiple environmental parameters influence the productivity and distributions of individual species, and their grouping together as communities and ecosystems (Cloern and Jassby 2012, Goberville, et al. 2015, Goberville, et al. 2016, Hannah 2015, Lomolino, et al. 2006, Whittaker 1975). In the terrestrial realm, many parameters such as soil composition, mineral nutrients, pH, water availability, oxygen solubility, the presence of a river or a lake, species sensitivity to frost or drought, influence biodiversity locally (Begon, et al. 2006, Hannah 2015). The heterogeneity of resources driven by the topography, land-use changes, historical processes may sometimes explain the patchy distribution of species (Guisan and Thuiller 2005). The vertical distribution of the quality and quantity of solar radiations in a forest (i.e. changes in canopy and albedo structure) affect species occurrence (Begon, Townsend and Harper 2006).

In the marine realm, nutrients and light limit phytoplankton production (Behrenfeld 2010, Sverdrup 1953). Both bathymetry and local spatial variability in bathymetry are key determinant of the marine pelagic biodiversity (Helaouët and Beaugrand 2007). Dissolved oxygen must remain high enough to support respiration (Goberville, et al. 2010). Mixed Layer Depth (MLD) is an important parameter for phytoplankton production and controls the spatial distribution of many plankton species (Longhurst 2007, Sverdrup 1953). Oceanic pH influences calcifying organisms such as coccolithophorids, foraminifers, corals and pteropods (Kroeker, et al. 2010, Orr, et al. 2005). Wind intensity affects prey-predator encounter rates (Rothschild and Osborn 1988) by its effects on oceanic turbulence, and nutrient supply rates by its effects on vertical mixing (Longhurst 2007). Wind direction, by its control of the distribution of some meroplankton species, might strongly affect recruitment of some benthic organisms (Jolly, et al. 2009). Light at the seabed is also an important factor for species depending on primary producers for food and for reproduction (e.g. coral reefs, mangroves, macro and microalgae).

The above lists are far from exhaustive, but show the complexity of pathways and types of control that the environment might exert on organisms and biocoenoses. Consequently, future versions of our framework should consider several environmental parameters simultaneously. However, we may find that the inclusion of additional parameters may not improve the model significantly because so many environmental parameters covary with temperature (e.g. ice, oxygen, some nutrients). Furthermore, data for many environmental parameters are not available with sufficient accuracy on a global scale and on a monthly basis. Hence, we have first focused on climatic parameters (temperature and total precipitation). Finally, our models cannot realistically implement biotic interactions in the construction of pseudo-communities on a global scale. Multiple examples suggest that they can be quite important in some ecosystems (Estes, et al. 2011, Hannah 2015, Kirby and Beaugrand 2009), however.

**Table S1.** **Correlations between simulated and observed species richness on land based on temperature and precipitation, temperature only and precipitation only.** All correlations were significant at the threshold of p<0.001. Values in bold are the highest correlations of the three simulations (precipitation and temperature together, precipitation only and temperature only). T: temperature. P: precipitation. The table shows that some taxonomic groups were more sensitive to just one of the climatic factors. For example, precipitation-based simulations explained better large-scale biodiversity patterns than temperature-based simulations for plants and amphibians.

| **Group** | **Correlation**  **(T & P)** | **Correlation**  **(T)** | **Correlation**  **(P)** | **Degree of freedom (n)** |
| --- | --- | --- | --- | --- |
| Plant | **0.7544** | 0.5317 | 0.6949 | 63105 |
| Amphibian | **0.7024** | 0.4731 | 0.6185 | 52994 |
| Reptile | 0.6938 | **0.7808** | 0.4956 | 84198 |
| Lizard and snake | 0.658 | **0.7469** | 0.4781 | 63105 |
| Turtle and crocodilian | **0.7521** | 0.7155 | 0.6294 | 84378 |
| Bird | **0.7712** | 0.6277 | 0.6535 | 76528 |
| Non-breeding bird | **0.7871** | 0.6361 | 0.6334 | 75600 |
| Breeding bird | **0.7534** | 0.5916 | 0.6609 | 76150 |
| Mammal | **0.7688** | 0.5855 | 0.6714 | 71183 |
| **Average correlation** | **0.7379** | **0.6321** | **0.6150** | - |

Table S2. **Comparison of total pseudo-species richness between domains and ecological zones.** ψ_1_: pool of niches. ψ _2_: number of niches that can potentially be present in a domain or an ecological zone. ψ _3_: percentage of niches that can potentially be present in a domain or an ecological zone. PS: pseudo-species. ψ _4_: mean number of pseudo-species per niche. ψ _5_: total number of pseudo-species. ψ _6,_ ψ _7,_ ψ _8_: median (ψ _6_), first (ψ _7_) and third (ψ _8_) quartiles of the area (km²) occupied by a pseudo-species. ψ _9_: percentage of the total area occupied by a pseudo-species. ψ _10_: seasonal stability in pseudo-species richness. T: temperature. P: Precipitation. The percentage value below T, P and T & P is the total number of niches randomly selected (see Materials and Methods).

|  | **Domain**  **or zone**  **(million km²)** | **Variable** | **Pool of niches**  **(ψ_1_)** | **ψ_2_** | **ψ _3_** | **ψ _4_** | **ψ _5_**  x 10^6^ | **ψ _6_**  **(ψ _7-_ ψ _8_)**  x 10^4^ | **ψ _9_** | **ψ _10_** |
| --- | --- | --- | --- | --- | --- | --- | --- | --- | --- | --- |
| **Land** | **Surface global**  **(146.52)** | T & P  (1%) | 73005 | 38990 | 53.40 | 20.91 | 81.53 | 2.2  (0.6-144.4) | 0.01 | 42.07 |
|  | **Surface global**  **(146.52)** | P (100%) | 72 | 52 | 72.22 | 23.07 | 0.001 | 2.0  (0.6-139.3) | 0.01 | 100.00 |
|  | **Surface global**  **(146.52)** | T  (25%) | 25349 | 25164 | 99.27 | 38.04 | 3.82 | 241.0  (168.6-319.6) | 1.64 | 45.35 |
| **Marine** | **Surface**  **Global**  **(355.44)** | T  (25%) | 25349 | 24112 | 95.12 | 13.17 | 1.27 | 1472.9  (781.7-2058.7) | 4.14 | 79.91 |
|  | **Surface neritic**  **(<200m)**  **(19.91)** | T  (25%) | 25349 | 24112 | 95.12 | 53.11 | 5.12 | 15.7  (12.1-22.1) | 0.78 | 42.61 |
|  | **Surface oceanic**  **(>200m)**  **(337.07)** | T  (25%) | 25349 | 23903 | 94.29 | 16.11 | 1.54 | 1164.1  (612.6-1623.2) | 3.45 | 80.55 |
|  | **Benthic global**  **(369.93)** | T  (25%) | 25349 | 24446 | 96.43 | 83.17 | 8.13 | 18.8  (11.4-37.3) | 0.05 | 98.63 |
|  | **Benthic neritic**  **(<200m)**  **(28.46)** | T  (25%) | 25349 | 24446 | 96.43 | 56.42 | 5.51 | 12.7  (8.8-18.4) | 0.44 | 69.31 |
|  | **Benthic oceanic**  **(>2000m)**  **(301.84)** | T  (25%) | 25349 | 22747 | 89.73 | 26.93 | 2.45 | 6.0  (1.4-18.8) | 0.02 | 99.42 |
|  | **Benthic shelf-edge**  **(200-2000m)**  **(35.99)** | T  (25%) | 25349 | 23600 | 93.10 | 90.65 | 8.56 | 7.5  (3.9-16.2) | 0.21 | 94.55 |

Figure S1. Modelled (red) and observed latitudinal biodiversity gradients (LBGs) for the terrestrial and marine realms. A. LBGs for the terrestrial realm. B. LBGs for the marine epipelagic realm. C. LBGs for the nerito-benthic (with light at the seabed) realm. D. LBGs for the neritic (nerito-pelagic and nerito-benthic) realm. Each value for a latitude represents the median of either observed or expected species richness. Black curves represent observed LBGs for taxonomic groups belonging to a specific realm (see Table 1).

**
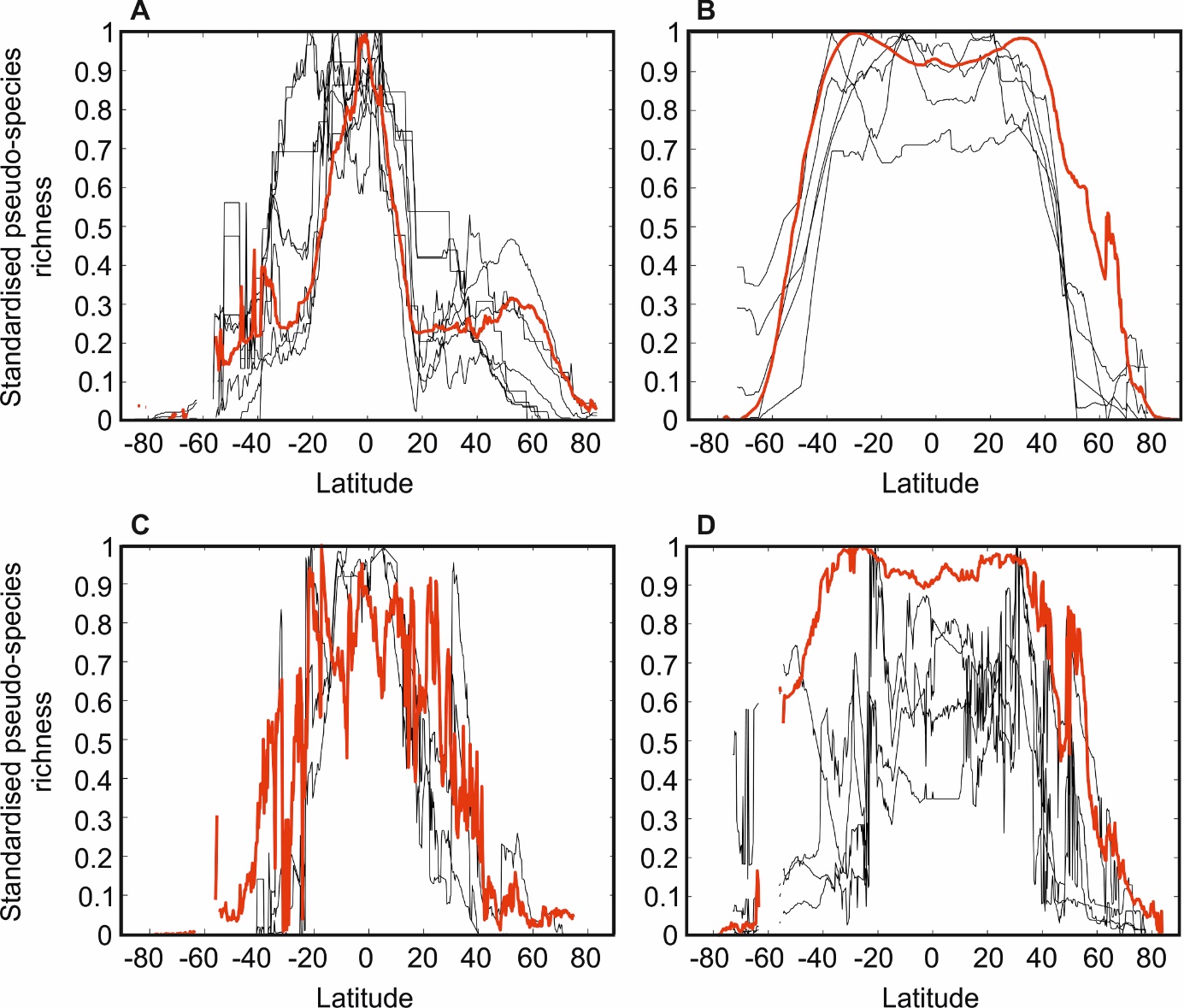
**

Figure S2. Latitudinal gradients in some climatic parameters over land (red) and oceanic (blue) areas. A. Latitudinal gradient in mean sea level pressure (mean SLP). B. Latitudinal gradient in mean downward solar radiation at surface. C. Latitudinal gradient in mean total precipitation.


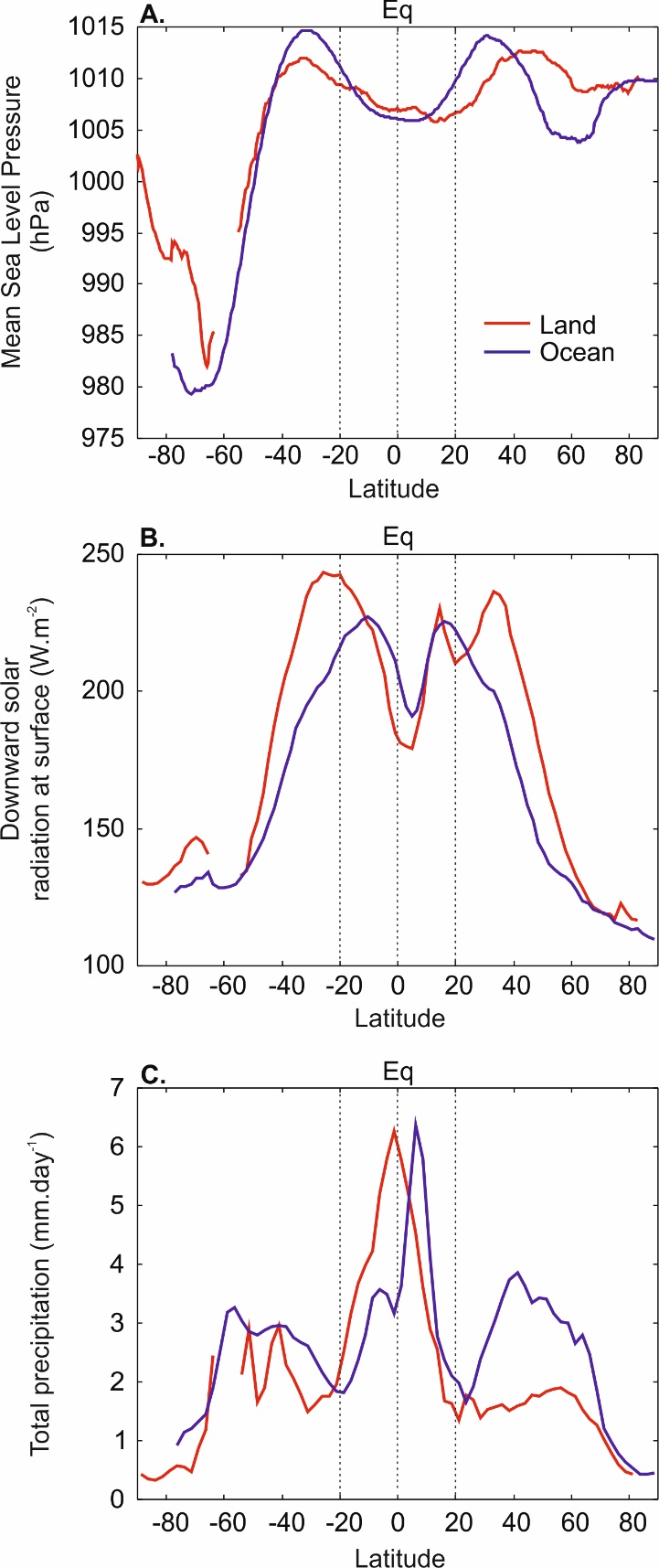


Figure S3. Latitudinal changes in mean bathymetry between (A) 0-200m, (B) 200-2000m and (C) above 2000m.

**
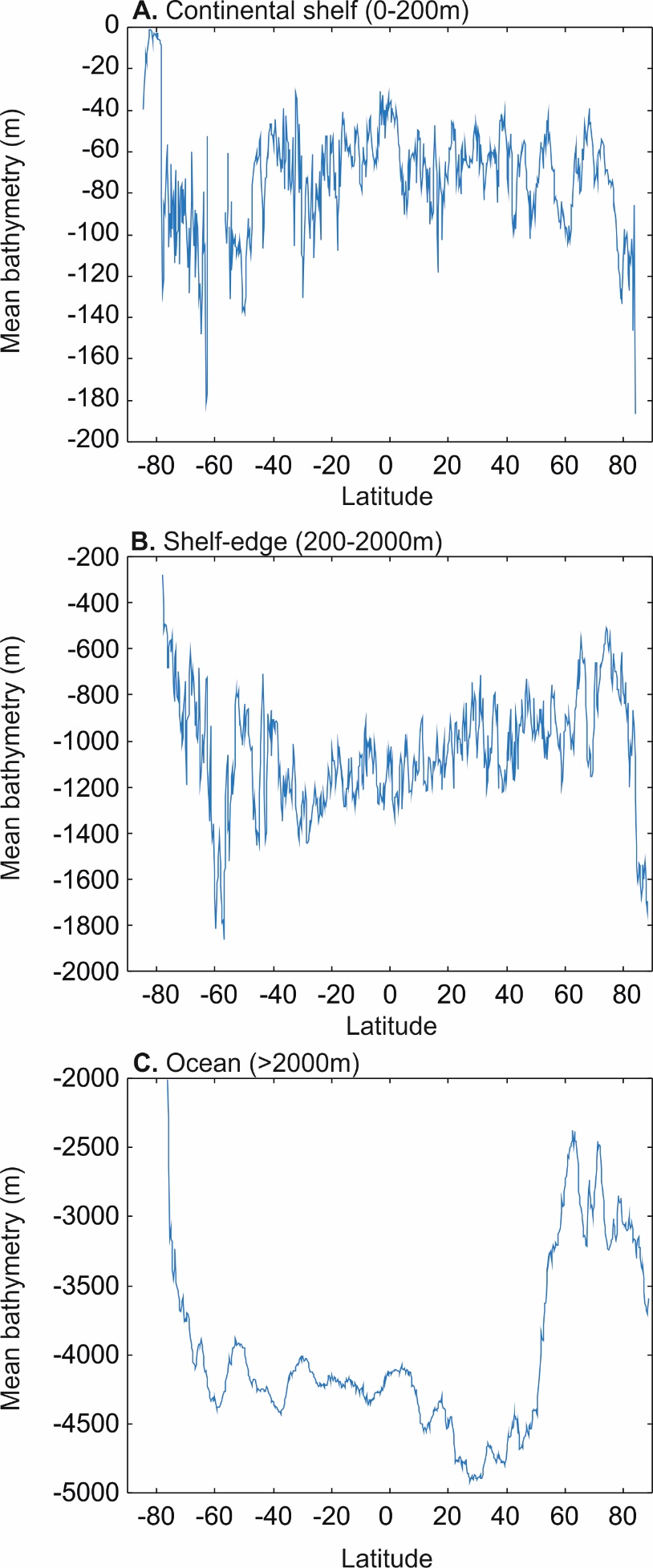
**

Figure S4. Examples of species spatial distribution that considered allopatric speciation. A different colour denotes a different species. A: Euryecious polar species. B. Stenoecious polar species. C: Euryecious temperate species. D. Stenoecious temperate species. E: Circumtropical species. F. Stenoecious tropical species. G: Euryecious tropical species. H. Equatorial tropical species.

**
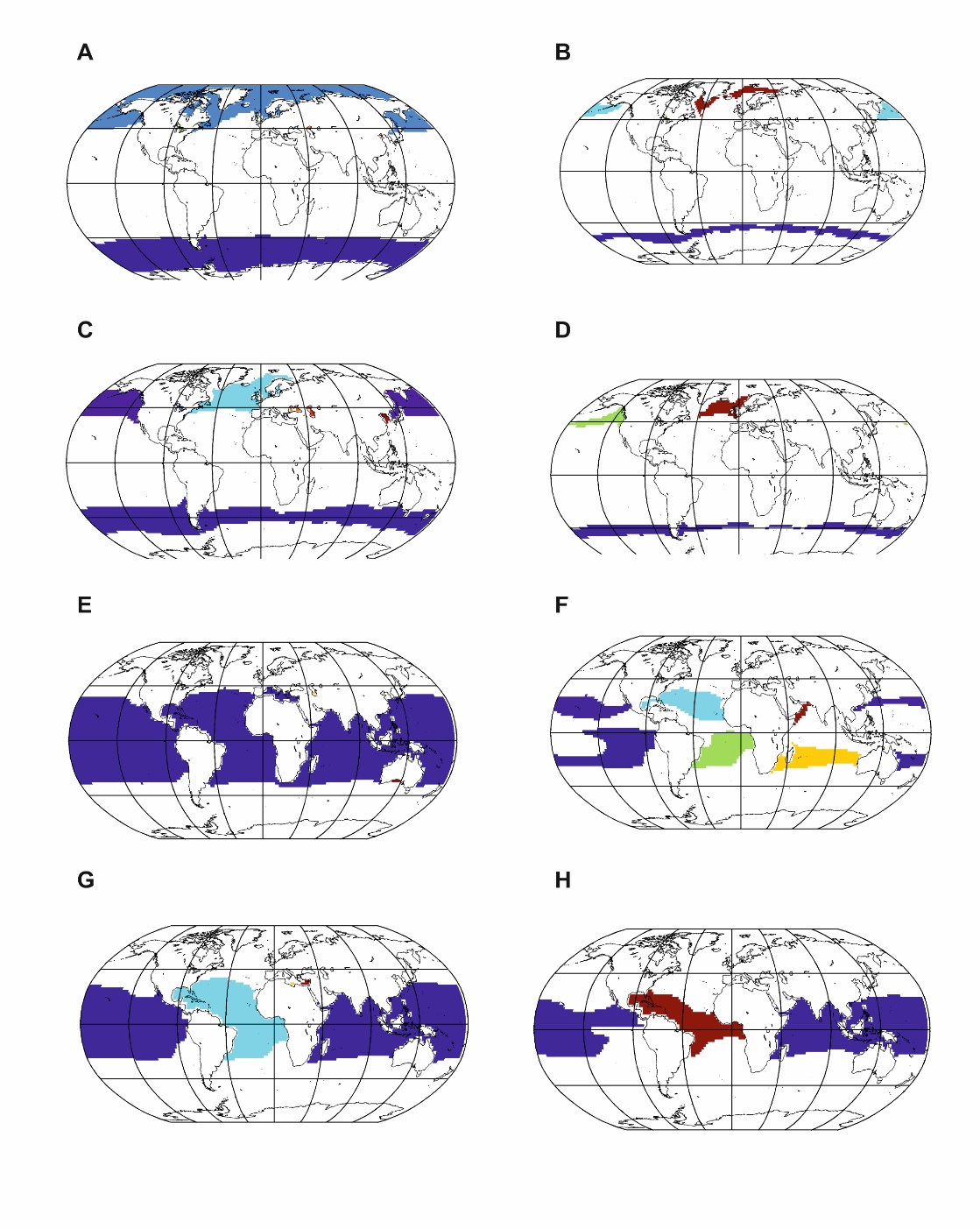
**

**References and notes**

Albouy, C., et al. 2012. Projected climate change and the changing biogeography of coastal Mediterranean fishes. - Journal of Biogeography 40: 534-547.

Araujo, M. B. and Guisan, A. 2006. Five (or so) challenges for species distribution modelling. - Journal of Biogeography 33: 1677-1688.

Bartlein, P. J., et al. 1986. Climatic response surfaces from pollen data for some eastern North American taxa. - Journal of Biogeography 13: 35-57.

Beaugrand, G., et al. 2002. Reorganisation of North Atlantic marine copepod biodiversity and climate. - Science 296: 1692-1694.

Beaugrand, G., et al. 2008. Causes and projections of abrupt climate-driven ecosystem shifts in the North Atlantic. - Ecology Letters 11: 1157-1168.

Beaugrand, G., et al. 2010. Marine biodiversity, ecosystem functioning and the carbon cycles. - Proceedings of the National Academy of Sciences of the USA 107: 10120-10124.

Beaugrand, G., et al. 2013. Applying the concept of the ecological niche and a macroecological approach to understand how climate influences zooplankton: advantages, assumptions, limitations and requirements. - Progress in Oceanography 111: 75-90.

Beaugrand, G., et al. 2013. Towards an understanding of the pattern of biodiversity in the oceans. - Global Ecology and Biogeography 22: 440–449.

Beaugrand, G., et al. 2014. Marine biological shifts and climate. - Proceedings of the Royal Society B: Biological Sciences 281: 20133350.

Beaugrand, G. 2015a. Theoretical basis for predicting climate-induced abrupt shifts in the oceans. - Philosophical Tansactions of the Royal Society B: Biological Sciences 370 20130264.

Beaugrand, G. 2015b. Marine biodiversity, climatic variability and global change. - Routledge.

Beaugrand, G., et al. 2015. Future vulnerability of marine biodiversity compared with contemporary and past changes. - Nature Climate Change 5: 695-701.

Beaugrand, G. and Kirby, R. R. 2016. Quasi-deterministic responses of marine species to climate change. - Climate Research 69: 117-128.

Beaugrand, G. and Kirby, R. R. 2018a. How do marine species respond to climate change? Theories and observations. - Annual Review of Marine Sciences 10: 169–197.

Beaugrand, G. and Kirby, R. R. 2018b. How do marine pelagic species respond to climate change? Theories and observations. - Annual Review of Marine Science 10: 169–197.

Beaugrand, G., et al. 2018. Marine biodiversity and the chessboard of life -Plos One 13: e0194006.

Begon, M., et al. 2006. Ecology. From individuals to ecosystems. - Blackwell Publishing.

Behrenfeld, M. J. 2010. Abandoning Sverdrup's critical depth hypothesis on phytoplankton blooms. - Ecology 91: 977-989.

Brown, J. H. 1984. On the relationship between abundance and distribution of species. - The American Naturalist 124: 255-279.

Brown, J. H., et al. 2004. Toward a metabolic theory of ecology. - Ecology 85: 1771-1789.

Chase, J. M. and Leibold, M. A. 2003. Ecological niches: linking classical and contemporary approches. - The University of Chicago.

Cheung, W. W. L., et al. 2008. Modelling present and climate_shifted distribution of marine fishes and invertebrates. - In: Fisheries Centre Research Reports, p. 72.

Cloern, J. E. and Jassby, A. D. 2012. Drivers of change in estuarine-coastal ecosystems: discoveries from four decades of study in San Francisco Bay. - Reviews of Geophysics 50: rg4001.

Elton, C. 1927. Animal ecology. - Sidgwick and Jackson.

Estes, J. A., et al. 2011. Trophic downgrading of Planet Earth. - Science 333: 301-306.

Gause, G. F. 1934. The struggle for coexistence. - MD: Williams and Wilkins.

Goberville, E., et al. 2010. Climate-driven changes in coastal marine systems of Western Europe. - Marine Ecology Progress Series 408: 129:147.

Goberville, E., et al. 2015. Uncertainties in species distribution projections and general circulation models. - Ecology and Evolution 5: 1100-1116.

Goberville, E., et al. 2016. Climate change and the ash dieback crisis. - Scientific Report 6: 35303.

Grinnell, J. 1917. The niche-relations of the California thrasher. - Auk 34: 427-433.

Guisan, A. and Thuiller, W. 2005. Predicting species distribution: offering more than simple habitat models. - Ecology Letters 8: 993-1009.

Hannah, L. 2015. Climate change biology. - Elsevier.

Helaouët, P. and Beaugrand, G. 2007. Macroecology of *Calanus finmarchicus* and *C. helgolandicus* in the North Atlantic Ocean and adjacent seas. - Marine Ecology Progress Series 345: 147-165.

Helaouët, P., et al. 2011. Macrophysiology of *Calanus finmarchicus* in the North Atlantic Ocean. - Progress in Oceanography 91: 217-228.

Hutchinson, G. E. 1957. Concluding remarks. - Cold Spring Harbor Symposium Quantitative Biology 22: 415-427.

Hutchinson, G. E. 1978. An introduction to population ecology. - Yale University Press.

Jolly, M. T., et al. 2009. Population genetics and hydrodynamic modeling of larval dispersal dissociate contemporary patterns of connectivity from historical expansion into European shelf seas in the polychaete *Pectinaria koreni* (Malmgren). - Limnology and Oceanography 54: 2089-2106.

Kirby, R. R. and Beaugrand, G. 2009. Trophic amplification of climate warming. - Proceedings of the Royal Society London B: Biological Sciences 276: 4095–4103.

Kroeker, K. J., et al. 2010. Meta-analysis reveals negative yet variable effects of ocean acidification on marine organisms. - Ecology Letters 13: 1419-1434.

Lenoir, S., et al. 2011. Modelled spatial distribution of marine fish and projected modifications in the North Atlantic Ocean. - Global Change Biology 17: 115-129.

Lomolino, M. V., et al. 2006. Biogeography. - Sinauer Associates, Inc.

Longhurst, A. 2007. Ecological geography of the sea. - Elsevier.

Orr, J. C., et al. 2005. Anthropogenic ocean acidification over the twenty-first century and its impact on calcifying organisms. - Nature 437: 681-686.

Pearman, P. B., et al. 2008. Prediction of plant species distributions across six millennia. - Ecology Letters 11: 357-369.

Pörtner, H. O. and Farrell, A. P. 2008. Physiology and climate change. - Science 322: 690-692.

Raybaud, V., et al. 2013. Decline in Kelp in West Europe and Climate. - PLOS One 8: e66044.

Rombouts, I., et al. 2009. Global latitudinal variations in marine copepod diversity and environmental factors. - Proceedings of the Royal Society B 276: 3053-3062.

Rombouts, I., et al. 2010. A multivariate approach to large-scale variation in marine planktonic copepod diversity and its environmental correlates. - Limnology and Oceanography 55: 2219-2229.

Rothschild, B. J. and Osborn, T. R. 1988. Small-scale turbulence and plankton contact rates. - Journal of Plankton Research 10: 465-474.

Roubicek, A. J., et al. 2010. Does the choice of climate baseline matter in ecological niche modelling? - Ecological Modelling 221: 2280-2286.

Rutherford, S., et al. 1999. Environmental controls on the geographic distribution of zooplankton diversity. - Nature 400: 749-753.

Sarmiento, J. L., et al. 2004. Response of ocean ecosystems to climate warming. - Global Biogeochemical Cycles 18: 1-23.

Schmidt-Nielsen, K. 1990. Animal physiology: adaptation and environment. - Cambridge University Press.

Stevens, G. S. 1989. The latitudinal gradient in geographic range: how so many species coexist in the tropics. - The American Naturalist 133: 240-256.

Sunagawa, S., et al. 2015. Structure and function of the global ocean microbiome. - Science 348:

Sunday, J. M., et al. 2012. Thermal tolerance and the global redistribution of animals. - Nature Climate Change 1-5.

Sverdrup, H. U. 1953. On conditions for the vernal blooming of phytoplankton. - Journal du Conseil Permanent International pour l'Exploitation de la Mer 18: 287-295.

Whittaker, R. H. 1975. Communities and ecosystems. - Macmillan.
